# Simulation of heterosis in a genome-scale metabolic network provides mechanistic explanations for increased biomass production rates in hybrid plants

**DOI:** 10.1038/s41540-019-0101-8

**Published:** 2019-07-18

**Authors:** Michael Vacher, Ian Small

**Affiliations:** 1 Australian Research Council Centre of Excellence in Plant Energy Biology, School of Molecular Sciences, The University of Western Australia, Crawley, WA 6009 Australia; 2 Present Address: Australian eHealth Research Centre, Commonwealth Scientific and Industrial Research Organisation, Floreat, WA 6014 Australia

**Keywords:** Computer modelling, Systems analysis

## Abstract

Heterosis, or hybrid vigour, is said to occur when F1 individuals exhibit increased performance for a number of traits compared to their parental lines. Improved traits can include increased size, better yield, faster development and a higher tolerance to pathogens or adverse conditions. The molecular basis for the phenomenon remains disputed, despite many decades of theorising and experimentation. In this study, we add a genetics layer to a constraint-based model of plant (*Arabidopsis*) primary metabolism and show that we can realistically reproduce and quantify heterosis in a highly complex trait (the rate of biomass production). The results demonstrate that additive effects coupled to the complex patterns of epistasis generated by a large metabolic network are sufficient to explain most or all the heterosis seen in typical F1 hybrids. Such models provide a simple approach to exploring and understanding heterosis and should assist in designing breeding strategies to exploit this phenomenon in the future.

## Simulation of heterosis in a metabolic network

Heterosis (hybrid vigour) plays a major role in many crops and livestock used for food production. However, the molecular basis for the increased biomass production in hybrids remains unclear. We propose a computational approach that integrates genetic constraints into a genome-scale metabolic model in order to simulate and better understand this phenomenon. The approach was able to reproduce the effects of selection pressure and heterosis. Analysing a large set of predicted metabolic fluxes in hybrid plants and their parents provided an unprecedented level of detail on the causes of heterosis in this simplified system. We could show that heterosis naturally emerges from multi-locus epistatic effects inherent across a complex biological network. Simple additive effects are sufficient to create significant heterosis without the need for specific biological mechanisms linked to heterozygosity.

## Introduction

As early as 1876, Charles Darwin provided the first scientific observations that interspecific hybrids tended to show more vigour than intraspecific hybrids.^1^ The phenomenon of heterosis has been recognised for over a century.^2^ Heterosis can be observed in both plants and animals, and presumably would occur in any diploid organism where such F1 hybrids can be obtained. Animal and plant breeders have benefited from hybrid vigour to produce increasingly more effective strains. In the USA, the use of hybrid maize has contributed to a dramatic six-fold increase in yield since the 1930s.^3^ Its benefits have stimulated extensive study into improving the design and selection of commercially valuable hybrids (reviewed in ref. ^4^). However, the molecular explanation for the phenomenon remains a topic of lively discussion. Early studies showed that heterosis was proportional to the genetic distance between two parents.^5–7^ More recent work has added considerable detail about how the genetic landscape of the parental lines might influence heterosis.^8,9^ Several competing but not mutually exclusive genetic models have been proposed in an attempt to provide some mechanistic explanation of the phenomenon.

The *dominance* hypothesis holds that independent sets of deleterious alleles accumulate over time and particularly during inbreeding of parental lines.^5,7^ In these conditions, dominant alleles originating from one parent would complement inferior alleles from the second parent, thus resulting in a phenotypic improvement. The *overdominance* hypothesis suggests the existence of allelic interactions that induce greater expression of heterozygous loci in hybrids.^10,11^ Neither of these hypotheses involve interactions between non-allelic loci. Additional hypotheses that do involve such interactions have been proposed. *Pseudo-overdominance*^11^ is the appearance of overdominance due to repulsion-phase linkage of dominant alleles.^4^ The effects of *epistasis* on heterosis are also becoming increasingly clear as the complexity of the biological networks underlying multigenic traits becomes better understood.^12^ Epistasis not only shapes which loci can express heterosis but it can also mimic overdominance.^13^ All of these mechanisms could, and probably do, contribute to heterosis. However, debate continues over which are the more important, because of the difficulty in experimentally distinguishing these intertwined genetic effects.

The potential complexity of the mechanisms contributing to heterosis has encouraged attempts to take a ‘systems’ approach to the topic, with large-scale molecular profiling to analyse gene expression and biochemical phenotypes.^14–23^ Such studies have provided much data but perhaps fewer clear explanations than might be hoped, with different studies reaching different conclusions about which molecular mechanisms predominate.

An alternative approach to treating such complexity is to attempt to model it mathematically or computationally. Modelling allows deliberate simplification of the system to make interpretation of the results easier, either by reducing the number of components, or their interactions, or both. Genetic models of heterosis have been studied for many years, but generally leave out downstream biological interactions that intervene in the complex interplay between genotype and phenotype. Only a few attempts have been made to bridge this gap and link genetic models of heterosis to biological networks that can simulate the extent of potential epistatic interactions between loci, and these have been limited to small-scale networks.^13,24^ We take this approach a step further by adding a simple genetics layer to a genome-scale model of plant primary metabolism. This combined model demonstrates plausible levels of heterosis under a set of realistic assumptions based on simple additive effects coupled to epistasis.

## Results

### Adding genetics to a model of metabolism

Standard descriptions of genome-scale metabolic networks do not generally incorporate any notion of genetic variability or heterozygosity, thus to study heterosis, an additional layer of genetic information is required. We modified a general model of *Arabidopsis* metabolism^25^ such that each reaction is assigned to a genetic locus, and each locus can be assigned a collection of alleles, allowing the simulation of diploid and polyploid individuals. The original metabolic model was designed for flux balance analysis, in which steady-state metabolic fluxes are calculated for each reaction under external metabolic constraints on the network (e.g. limitations on the rate of photosynthesis). In general, the metabolic fluxes are optimised to maximise the desired output, for example, biomass production. In our additions to this model, we added internal genetic constraints. Each allele consists of a constraint that can be applied to a reaction in order to confine its metabolic flux. The rationale behind this is that the genetic information determines the level of expression and thus the activity of the enzymes in the metabolic network. In the simulations described here, this genetic information acts in *cis*, that is, we have included no *trans*-acting regulatory factors. This simplification does not prevent heterosis appearing in the simulations because many cross-locus interactions are already provided by the metabolic network.

The constraint attached to each allele was chosen randomly, as follows. First, the generic model was optimised for the maximisation of biomass production (using flux balance analysis), resulting in a reference flux distribution (RFD). Flux variability analysis (FVA)^26^ was then performed on the network to calculate an envelope of alternate fluxes for each reaction that could maintain optimal biomass production. Allele-specific constraints were then generated using the FVA as a guide (such that all alleles had constraints within their allowable flux range, simulating recurrent natural selection for efficient biomass production). Each individual within the population was assigned two randomly chosen alleles at each locus (to simulate a heterozygous individual). In simulated crosses, alleles at each locus were transmitted randomly, independently and unaltered; that is, we did not attempt to simulate genetic linkage, mutation or any epigenetic effects. By default, we assumed that the genetic effects of multiple alleles are additive, that is, we took the average genetic constraints from the alleles at one locus to set a single constraint for each reaction.

Prior to any further calculations, the flux constraints assigned to each individual were normalised such that the sum total of all constraints corresponds to the sum total of all the fluxes in the RFD. Thus, the total amount of ‘enzyme’ that any individual can produce is fixed in our simulations. This constraint was introduced to avoid trivial results where rate of biomass production is simply dependent on the total flux capacity (equivalent to the trivial real-life observation that larger individuals can sustain higher rates of biomass production). Holding total flux capacity constant forces any changes in rate of biomass production in the simulations to be dependent in changes in metabolic efficiency—producing more interesting outcomes. The biomass production network was then optimised for each individual using the constraints specific to that individual. Thus, for each reaction in the network, we obtain four flux values (in the case of a diploid individual): the two potential flux constraints encoded by the alleles at the locus, the genetic flux constraint calculated from these allele values (and subsequent normalisation) that was used during flux balance analysis (henceforth referred to as the enzyme capacity) and the calculated flux through the reaction following optimisation of biomass production (henceforth referred to as the computed flux).

### Effects of selection on inbreeding and heterosis

**Fig. 1:**
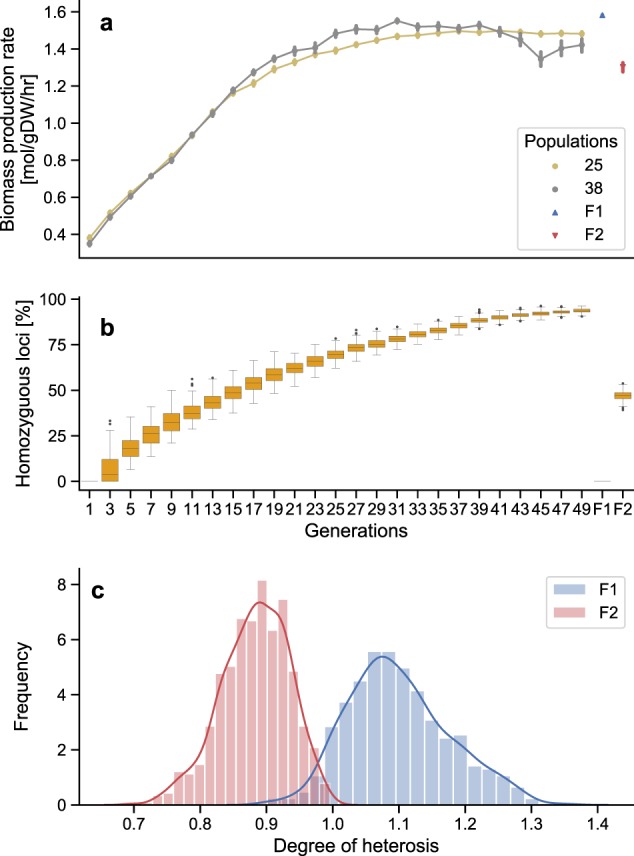
Selection for inbred populations and heterosis in crosses between them. **a** Starting with forty initial independent populations (two of which are shown here, the others in Supplementary Fig. S1), we simulated the effect of selection pressure over 50 generations. Each generation contains 500 individuals, from which the top 5% (individuals having the highest biomass production rate) were selected and used as parents for producing the next generation. After 50 generations, the two inbred lines were crossed to produce the F1 and subsequently the F2 populations. The F1 population attained ~34% of the maximum possible biomass production rate. **a**, **b** share the same horizontal axis. **b** The accumulation of homozygous alleles within these populations. After 50 generations the inbreds are homozygous at over 90% of their loci, whereas F1 individuals are 0% homozygous and the F2 individuals 50% homozygous. **c** Distribution of observed heterosis in 780 simulated crosses between the 40 inbred populations. Here we are defining heterosis as the ratio between the rate of biomass production in the F1 individual and the average of the rates in the two parents (i.e. mid-point heterosis). Values are the mean of 500 F1 or F2 individuals for each cross

To model the genetic composition of the inbred lines used in conventional breeding programs, we simulated the effect of selection by inbreeding 40 independent heterozygous populations over 50 generations (Supplementary Fig. S1), of which two examples are shown in Fig. 1a. At each generation, the best-performing individuals within each population (judged by rate of biomass production) were selected and crossed together to generate the following generation of increasingly inbred individuals. Over the first 30 generations, we observed an improvement in the mean biomass production rate. At the same time, the homozygosity of the population increased (Fig. 1b). In further generations, gains in the rate of biomass production slowed. These general trends were consistent between the 40 independent populations (and in multiple repeats of this experiment). After 50 generations, when mean homozygosity exceeded 90%, crossing individuals from two inbred populations produced heterozygous F1 hybrid populations showing heterosis for the rate of biomass production (Fig. 1a). Selfing the F1 populations produced F2 generations with a lower average rate of biomass production, even lower on average than that of the parental inbreds (Fig. 1c).

### Diverse routes to heterosis

**Fig. 2:**
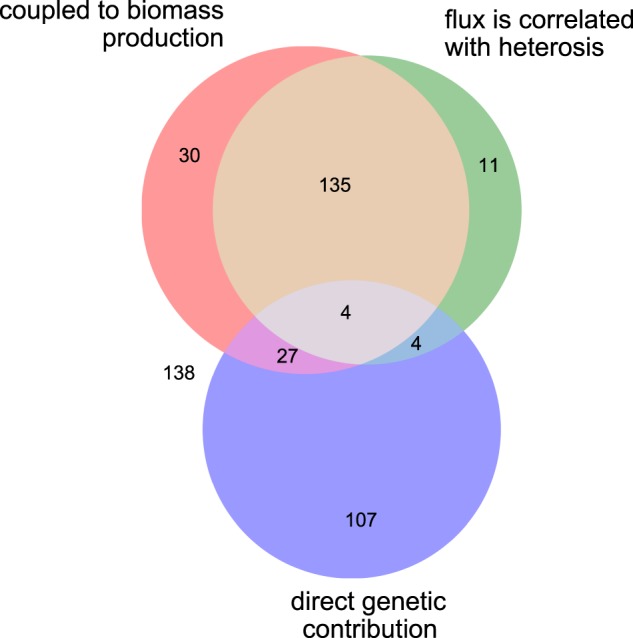
Venn diagram showing sets of reactions that contribute to biomass production and/or heterosis. Of the 456 reactions operating in the network, about one-third are coupled to biomass production, or their flux is correlated with heterosis in at least one F1 population, or alleles at the corresponding locus can be shown to directly contribute to heterosis. However, although the overlap between biomass coupling and flux correlation with heterosis is high, most direct genetic contributions to heterosis come from loci corresponding to uncoupled and uncorrelated reactions. The data on which the diagram is based are summarised in Table S1. The thresholds used to establish the set boundaries were |*r*| >0.9 (for correlations) and variance >1e − 8 for the contribution to heterosis

Each individual simulation corresponds to a linear optimisation problem where an objective function (production of biomass) is maximised. The computed flux through this objective function provides a highly multigenic trait indicative of overall performance. Detailed analysis of the rest of the metabolic network provides useful information regarding which reactions are contributing to heterosis. In order to identify reactions contributing to the increased performance in the F1, for each F1 population we calculated the correlation coefficient between the magnitude of heterosis in each F1 individual and the computed flux for each reaction in the network (Supplementary Table S1). A total of 154 reactions (~34% of the total) were strongly correlated with heterosis (|*r*| > 0.90) in at least one F1 population. These reactions included both internal reactions and transporters associated with the production of biomass components. Thus, as would be expected, heterosis in these simulations appears to be due to increased metabolic flux through pathways relevant to biomass production. This can be shown formally by calculating the coupling between each reaction and the production of biomass.^27^ Indeed, 90% of the highly correlated reactions were found to be coupled with biomass production (Fig. 2). Nevertheless, not all biomass-coupled reactions contribute equally to heterosis.

### Single vs. multi-locus contributions to heterosis

**Fig. 3:**
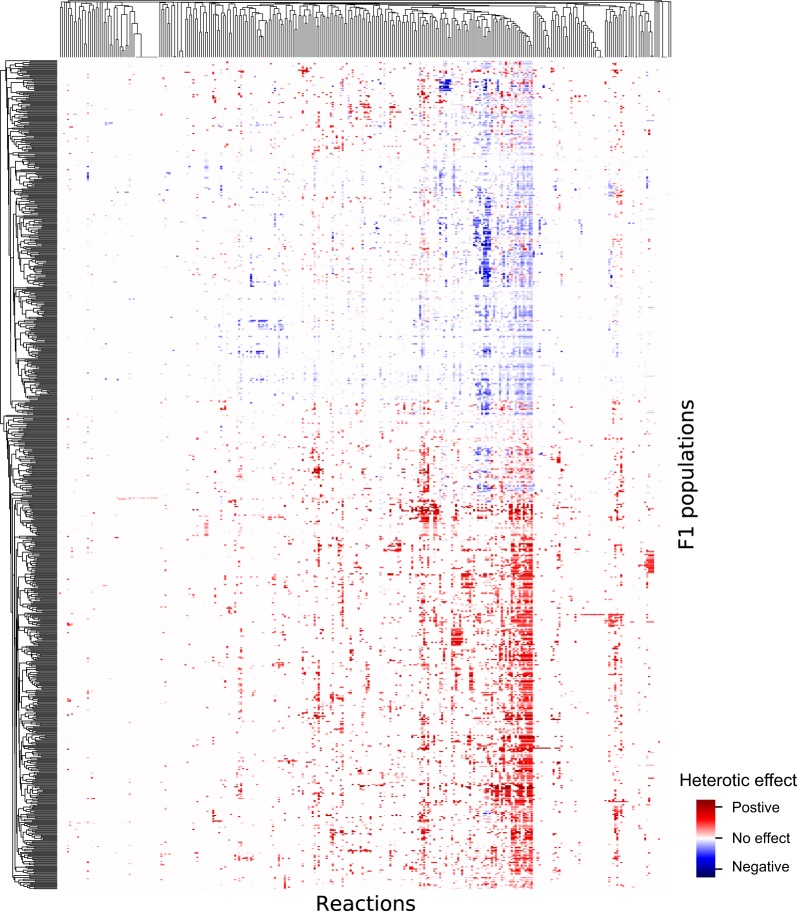
The contribution of individual metabolic reactions to heterosis. The heatmap depicts the contribution of each reaction in the network to heterosis in 780 F1 populations (the same populations as described in Fig. 1c and Supplementary Fig. S1). The contribution was calculated as the effect of rendering the locus homozygous for each parental allele on the rate of biomass production in the hybrid. Effects were considered ‘positive’ if rendering a locus homozygous resulted in an increase in the rate of the biomass production and negative if it resulted in a lower rate of biomass production. Contributions were summed across all 500 individuals in each F1 population. The full table of values with the names of the reactions are given in Supplementary Table S1. The red symbols above the heatmap indicate the reactions coupled to biomass production

**Fig. 4:**
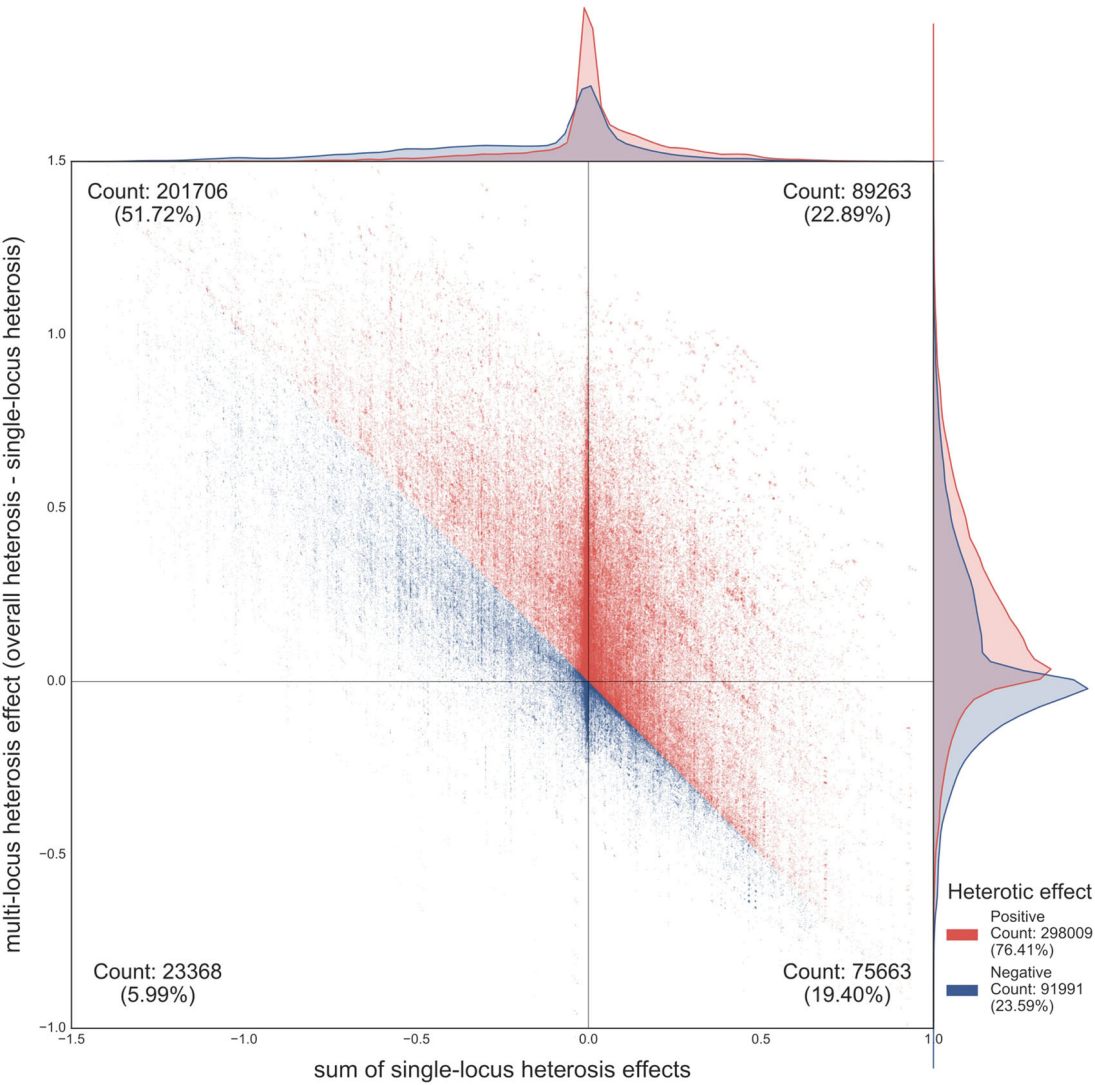
The effect of single and multi-locus contributions to heterosis. The scatter plot shows all 390,000 individuals, indicating the relationship between the sum of all individual locus effects on heterosis (*x*-axis) and multi-locus effects (*y*-axis). Multi-locus effects were calculated as the difference between overall heterosis and the sum of all individual locus effects. The distributions of the two variables are shown above and to the right of the plot. Individuals in red show positive heterosis, that is, a rate of biomass production greater than the average of the two parents

To examine the contribution of different reactions in the network to heterosis in more detail, we systematically altered alleles in the F1 individuals to render them homozygous for one or other parental allele at each locus in turn, and recalculated the computed flux for biomass production. If the recalculated flux was lower than that of the original F1 individual, then this revealed the direct contribution of the altered parental allele to heterosis. Figure 3 shows a heatmap depicting the direct contribution of all loci calculated in this way. The results indicate that different loci contribute to heterosis in different crosses, as one might expect. However, the patterns are not entirely random; some loci contribute much more to heterosis than others when viewed across many independent crosses. Thirty-one per cent of the reactions showed a strong direct contribution to heterosis in at least one F1 population. Surprisingly (at first sight), the loci at which direct contributions are found do not generally correspond to reactions coupled to biomass production or whose fluxes correlate with heterosis (Fig. 2). This prompted us to look for, and quantify, indirect effects. The direct contributions of all loci can be summed to give the heterosis arising only from single-locus effects. This in turn can be subtracted from the overall heterosis, leaving the part due to multi-locus effects. The comparison between these two components of heterosis (Fig. 4 and Supplementary Fig. S2) demonstrates the overriding importance of multi-locus interactions in these simulations. In ~70% of individuals, multi-locus effects make a larger contribution to heterosis than all the single-locus effects combined, and 40% of individuals show positive heterosis (owing to multi-locus effects), even though the sum of single-locus effects is negative (individuals coloured in red in the top left quadrant of Fig. 4).

### Hybrids make more efficient use of resources

**Fig. 5:**
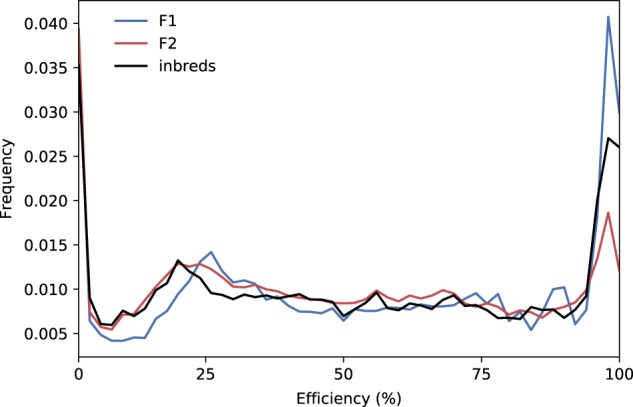
Variation of metabolic efficiency between inbreds, F1 and F2. The graph displays the metabolic efficiency for a collection of 390,000 F1 hybrids, their parents and the subsequent F2 population. Efficiency is calculated as the proportion of the flux capacity for each reaction that is actually used (computed flux/genetic flux constraint). A higher proportion of reactions show high efficiencies (over 95%) in F1 hybrids than in their inbred parents or in the F2 individuals

The efficiency of a reaction in our networks can be defined as the ratio between the computed flux and the enzyme capacity of the reaction (set by the genetic constraints). This represents the proportion of synthesised enzyme that is actually active. Figure 5 shows that when comparing F1 hybrids to their parents, there is a distinct improvement in reaction efficiencies—a higher metabolic flux is maintained with equal investment in enzyme synthesis. In particular, there is a sharp increase in the number of reactions operating at near maximal efficiency. This increase in efficiency underlies heterosis. These efficiency gains are lost in the F2 generation, when the efficiencies return to the level seen in the inbred parents (or even lower).

### Biochemical mechanism: the relaxation of metabolic bottlenecks

**Fig. 6:**
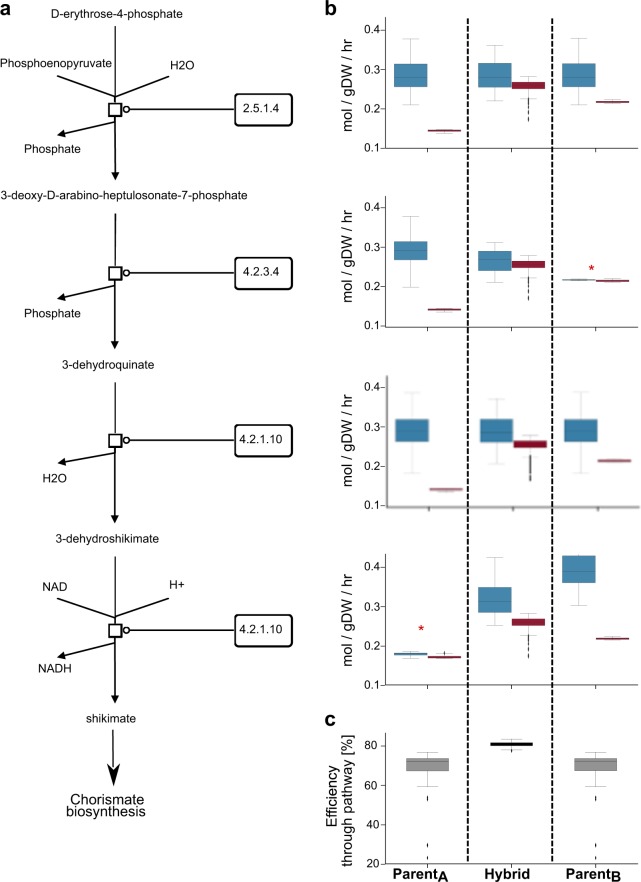
Multi-locus relaxation of bottlenecks in the chorismate pathway in an F1 hybrid. The right side of the figure represents a section of the chorismate biosynthetic pathway. On the left, the boxplots show the differences between enzyme capacity and computed flux for these four steps in the pathway. At the bottom, the boxplots show the efficiency of the pathway in the three populations. The data are from a collection of F1 hybrids and their parents (Parent_A_, Parent_B_). In the inbred parents, the fluxes through two reactions (EC 4.2.3.4 and EC 4.2.1.10) are limited by particularly low enzyme capacities. These bottlenecks reduce the activity of the upstream and downstream reactions. In the resulting F1 hybrids, the additive effect of the two alleles partially complements each of the two bottlenecks, resulting in a greater average efficiency for the pathway as a whole. In this simple example, relaxation of the metabolic bottleneck primarily only involved two loci; in most cases, the interactions are more complicated to unravel

The presence of many reactions in the network operating at below 100% efficiency implies the presence of metabolic bottlenecks. These bottlenecks are imposed by the direct genetic constraint on certain metabolic steps. However, they also impact the rest of the network by forcing upstream or downstream reactions to operate at rates below their maximal capacities. These bottlenecks are partially relaxed in F1 hybrids, as illustrated by the reactions whose efficiency improves. An example provided in Fig. 6 shows how multi-locus effects obtained by crossing parental lines can relax the constraints imposed by specific local bottlenecks, allowing a higher metabolic activity through the whole pathway. The arrangement of these bottlenecks can be due to random genetic differences in the original populations or due to selection for different biomass production strategies within the two parental inbred lines.

### ‘Fixing’ heterosis by recurrent selection

**Fig. 7:**
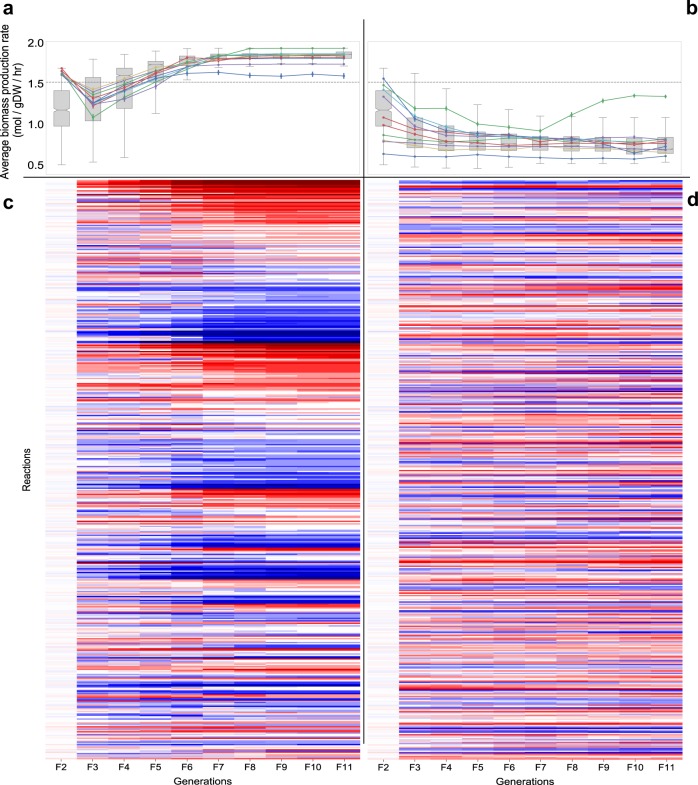
Selection of new inbred lines derived from F1 hybrids. An F1 individual exhibiting a typical degree of hybrid vigour was selected and used to generate a collection of 1000 F2 individuals. Twenty F3 populations were generated by selecting and selfing 20 F2 individuals; 10 of these were chosen at random, and 10 were selected for high biomass production rates. Each of the F3 populations consisted of 500 individuals from which 2% were selected (either at random, or on the basis of high biomass production rate) and selfed to produce 500 new individuals in each subsequent generation. The process was repeated until the F11 generation was reached. The left side shows biomass production rates and allele frequencies for the populations where the best-performing individuals were selected at each generation. The right side shows biomass production rates and allele frequencies for the populations where the individuals were selected at random from each generation. Columns show data from left to right for generations F2–F11. The top panels show the distribution of biomass production rates in each generation; the dashed line corresponds to the biomass production rate of the original F1 individual. In the heatmaps the intensity of the colours indicates allele frequencies in the population at each locus (red = parent_A; blue = parent_B)

It is evident that in the context analysed here, heterosis is largely not dependent on heterozygosity at any locus (i.e. mixing of parental alleles at one locus), but rather on epistatic effects across two or more loci. If this is the case, then it should be possible to ‘fix’ the improvements obtained in the F1 generation by selecting for the optimal combinations of parental alleles over subsequent generations. Therefore, we continued to select for biomass production in the F2 and ensuing generations produced by selfing (Fig. 7). The depression in the rate of biomass production in the F2 was rapidly reversed within a few generations, and from about the F5 generation, the rate exceeded from that observed in the F1. After approximately six generations, the new ‘inbred’ lines produced were nearing complete homozygosity while preserving rates of biomass production significantly higher than in the original F1.

## Discussion

Despite substantial efforts to define a universal theory explaining the phenomenon, the understanding of the molecular basis for heterosis has remained elusive. By reproducing and explaining several, if not all, aspects of heterosis, the modelling approach presented in this study can shed some light on this long-standing question. The models reproduced the progressive gain in performance and reduction in heterozygosity under intense selection over several generations and the subsequent plateau as the best alleles become fixed in the population.^28,29^ The models also demonstrated biologically plausible levels of heterosis, averaging around 10% of increased metabolic flux, which is in the range of what is commonly observed when crossing typical inbreds. The models also reproduced the collapse of heterosis in the F2 generation. Inspection of the reaction fluxes in the models provides a rational explanation for the mechanisms responsible for the observed improvement in hybrid F1 plants and allows the investigation and quantification of the contribution of different aspects of the genetic models.

Although the results we obtain appear to mimic heterosis, an obvious question is whether the models we describe accurately reflect a significant component of the processes underpinning heterosis in living organisms. Our models are necessarily greatly simplified, but we believe they do reproduce the most salient features of the genetics of heterosis. The results of any computational model are dictated by the choices made in its design; we believe we have made sensible and even conservative decisions in the choices of the metabolic and genetic models, and in the structure of the populations and the selection regime. As an example of a conservative choice, we used FVA analysis to set the range of the initial alleles within the population (equivalent to restricting its genetic diversity). Without using FVA to restrict the choice of starting alleles, individuals could contain strongly sub-optimal alleles (i.e. would be effectively ‘mutants’ in a genetic sense). We felt this would not be a good starting point for simulating heterosis in natural or breeding populations where recurrent selection will have eliminated almost all strongly sub-optimal alleles even prior to the creation of inbred lines. By reducing the ‘genetic variability’ in the starting populations, the use of FVA to set the bounds reduces the magnitude of the potential heterosis, but results in a more plausible simulation.

An important finding is that simple additive genetic effects are entirely sufficient to provide significant levels of heterosis without any requirement for dominant or overdominant genetic effects (by this we mean non-additive interactions between alleles at the same locus). Most experimental observations of gene expression in F1 hybrids have found that additive expression is by far the most common case.^15,17,21^ This, of course, does not rule out that dominance or overdominance might play important roles in specific instances, and this is supported by genetic studies specifically looking for loci underlying heterotic effects.^18,23,30^ Epistatic effects are hard to fully account for in such genetic studies if they involve multiple loci. Similarly, previous attempts to include epistasy in computational models of heterosis have by and large been limited to effects between two or only a few loci (e.g.^31^ which underestimates the interconnectivity in most biological contexts). The importance of epistasy and biological networks in shaping heterosis has been discussed at length^4,12^ but has been difficult to examine in practice. The originality of connecting a simple genetic model to a large metabolic network, as we have done, is in the capture of the complex epistatic effects created by the topology of the network. It is clear that in our simulations, effects involving multiple loci far outweigh the effects of any individual locus, or even of all individual effects summed together. Models such as those we describe should make it much easier to investigate the contribution of epistasy to heterosis.

If inter-locus interactions are more important than intra-locus interactions for heterosis (and in our models this is certainly the case), then the prediction is that the gains in the F1 generation can be fixed in homozygous inbreds that preserve the best parental allele combinations at different loci. We showed that in the models this is relatively simple to achieve by recurrent selection over a few generations. This is greatly facilitated in our simplified system by the lack of any genetic linkage that would interfere with the reassortment of parental alleles. Nevertheless, the results recapitulate a study in *Arabidopsis*, which showed that the improved phenotypic traits obtained in an F1 hybrid could be stabilised through the generation of the so-called ‘hybrid mimic’ lines.^32^

Despite the good match of the model outputs to experiment observations of heterosis, there are many ways in which these models could be improved, or at least extra complexities could be added that may be needed to more finely simulate specific biological contexts. Our model did not incorporate any notion of genetic linkage, allowing alleles to be inherited completely independently, which would lead to unrealistic rates of allele segregation and selection, but should not greatly affect the conclusions we draw here. More importantly, the model is lacking several layers of network complexity: notably there are no ‘regulatory genes’, and the metabolic model similarly lacks any feedback loops or metabolic regulation. These deficiencies should not alter the main concepts and conclusions demonstrated in this study, but may make it more difficult to apply such models for real-world purposes (such as predicting the levels of heterosis that would be obtained in specific crosses, or predicting ideal selection pathways to obtain inbreds for maximising heterosis). Nevertheless, we believe that this would be an interesting and rewarding direction to take this research in the future, as promising results in other studies have been achieved even without the benefit of explicit models of molecular interactions.^33^ By allowing a system-wide view, computational models provide valuable insights into complex biological processes or phenomena such as heterosis. Our models provide an approach for rigorously quantifying various contributions to heterosis by different genetic or metabolic processes. We hope that such models can ultimately be applied to improve the rational design of breeding and selection programs to maximise heterosis for agronomically relevant traits.

## Methods

### Metabolic network and simulations

We used the metabolic network of *Arabidopsis thaliana* presented in ref. ^25^ The model consists of 549 reactions, 407 metabolites and 6 sub-cellular compartments. The composition and topology of the network were not altered, except for the integration of allele-specific constraints as described below. We used a parsimonious flux balance analysis (pFBA) to simulate growth in all the experiments. This method uses a two-step optimisation in which the growth rate is optimised using traditional flux balance analysis, followed by the minimisation of the total flux through all the reactions.^34^ Minimising the total flux limits the number of possible solutions and improves the predicted flux distribution by representing an efficient enzyme usage within the network. The original model provides three different biomass compositions: carbon-limiting, nitrogen-limiting, and optimal growth conditions (under which biomass accumulation is only limited by photon flux into photosynthesis). The latter was systematically used as the objective function in our simulations. Given the stoichiometric matrix of the network (*S*), flux balance analysis computes the vector of fluxes *v*, using the assumption that the system is at steady state:

1 $${\mathrm{Maximise}}\quad v_{\mathrm{biomass}} = \mathop {\sum}\nolimits_i {c_iv_i = {c} \cdot {v}}$$

2 $${\mathrm{subject}}\,{\mathrm{to}}\quad {S} \cdot {v} = 0$$

3 $${\mathrm{and}}\quad \alpha _j \le v_j \le \beta _j.$$

In order to minimise the total flux, all the reversible reactions are split into two irreversible reactions, resulting in the stoichiometric matrix *S*_irrev_. Thus, each reaction is constrained to carry a positive flux and the total flux is minimised subject to the optimal biomass production rate:

4 $${\mathrm{Minimize}}\quad \mathop {\sum}\nolimits_j {v_{{\mathrm{irrev}},j}}$$

5 $${\mathrm{subject}}\,{\mathrm{to}}\quad {S}_{\mathrm{irrev}} \cdot {v}_{\mathrm{irrev}} = 0$$

6 $${\mathrm{and}}\quad v_{\mathrm{irrev,biomass}} = v_{\mathrm{biomass}}$$

7 $${\mathrm{and}}\quad 0 \le v_{{\mathrm{irrev}},j} \le \beta _j.$$

### Generating allele-specific constraints

On the initial parents, metabolic constraints attached to each allele were generated as follows:

1. First, a pFBA was performed on the original model, resulting in an RFD. The analysis was set up to maximise the flux through the objective function (biomass) while minimising the sum of all the fluxes in order to limit futile cycles.
2. FVA was used to compute the maximum and minimum fluxes for each reaction that maintain a minimum fraction (95%) of the optimum biomass production rate. Alternative FVA minimum fractions (90% and 99%) were also tested, the results are available in Supplementary Fig. S1.
3. Each reaction was attributed a pair of alleles (to simulate a diploid organism). In this context, an allele corresponds to a random variable following the discrete uniform distribution over the set of possible fluxes within the FVA range of a given reaction.
4. Finally, reactions were constrained by assigning numerical values their upper or lower bound (depending on the reaction directionality, which was taken from the RFD). Exchange reactions and biomass reactions were left unconstrained in all the simulations. For all the other reactions, the constraint corresponds to the average of its allele values, normalised such as the sum of the constraints across all loci is equal or lower than the sum of all the fluxes in the RFD. The normalisation ensures that all the individuals have a similar metabolic capacity.

### Quantifying heterosis

The degree of heterosis of an F1 hybrid was defined as its biomass production rate divided by the average biomass production of the two parents, that is, mid-parent heterosis.

### Simulated crosses

To simulate the inheritance of genetic material, one allele from each parent was randomly selected for each reaction. The new pair of alleles was associated with the corresponding reaction in the resulting network. The reaction bounds were then constrained and normalised as described previously.

### Selection over multiple generations

To simulate the effect of selection pressure, individuals were ranked based on their biomass production rate and the top 5% were selected. These best-performing individuals were then used as parents to produce the following generation. Each generation consisted of 500 individuals. This process was repeated until the number of desired generations was reached. During the production of these increasingly inbred lines, selfing was prevented (a newly created individual is the result of a crossing between two distinct parents from the same line).

### Production of F1 and F2 hybrids

F1 individuals were obtained by randomly sampling 30% of the inbreds from two independent parental lines and crossing them together until the number of desired F1 hybrids was reached (in general, 500 individuals). The two parents were always from distinct populations; however, several F1 individuals could come from the same combination of parents. Such siblings will contain different combinations of alleles as the parents are not completely homozygous. The F2 generation was generated in a similar manner; 30% of the individuals from an F1 population were randomly selected and crossed with each other. In our implementation, an F2 individual always results from a cross between two distinct F1 parents (no selfing allowed) from the same population. To maximise the diversity of F1 and F2 hybrids, we used all possible combinations of crosses between our 40 inbred populations, resulting in 780 independent F1 and F2 populations containing 390,000 individuals in each generation.

### Design and implementation of the simulation workflow

The simulation of the metabolism of many individuals over multiple generations requires significant computing resources. To avoid scaling problems, the workflow was designed for parallel high-performance computing. We used a ‘divide and conquer’ design paradigm to break the computing tasks into single sub-problems corresponding the model optimisations. The entire workflow was written in Python. The simulations were performed using the COnstraint-Based Reconstruction and Analysis (COBRA) Python package^35^ and the GNU Linear Programming Kit (http://www.gnu.org/software/glpk).

### Reporting summary

Further information on research design is available in the Nature Research Reporting Summary linked to this article.

## Supplementary information

Supplementary Information

Reporting Summary

## Data Availability

The scripts required to produce the results presented in this study are available at the following address: https://bitbucket.org/mvacher/heterosis_manuscript.

## Supplementary information

**Supplementary information** accompanies the paper on the *npj Systems Biology and Applications* website (10.1038/s41540-019-0101-8).
