# Supplementary material for: Simulation of heterosis in a genome-scale metabolic network provides mechanistic explanations for increased biomass production rates in hybrid plants
Source: NPJ Syst Biol Appl. 2019 Jul 18;5:24. doi: 10.1038/s41540-019-0101-8 (PMC6639380; doi:10.1038/s41540-019-0101-8)
Supplement: Supplementary file 1 — Supplementary Information [file 41540_2019_101_MOESM1_ESM.pdf]

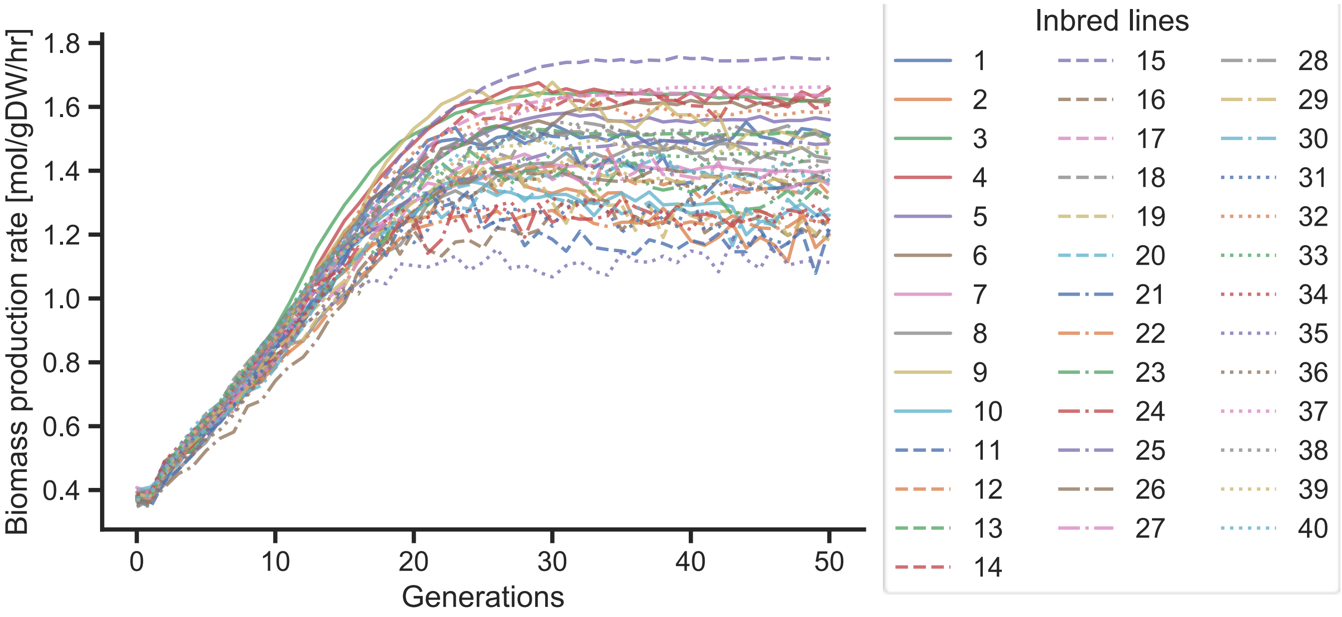

Supplementary Figure S1.

Starting with 40 initial collection of 25 parents (representing independent populations), we simulated the effect of selection pressure over 50 generations. Each generation contains 500 individuals from which the top 5% (individuals having the highest biomass production rate) were selected and used as parents for producing the next generation.

Supplementary Table S1.
The table summarises the data for all the metabolic reactions in the F1 hybrids.

sbml_id	reference_flux	pathways	coupled	correlated_in_n_populations	max_correlation	effect_variance	effect_max	contribute_in_n_populations	efficiency_median	efficiency_max
**2IPMS_h**	0.806508612	amino acid synthesis	TRUE	0	0.897217897	1E-09	0.00019381	0	67.21021854	92.83958863
**3IPMDA1_h**	0.806508612	amino acid synthesis	TRUE	1	0.94976327	4.86E-10	0.00019381	0	63.48893063	94.56142943
**3IPMDA2_h**	0.806508612	amino acid synthesis	TRUE	1	0.946589718	4.51E-10	0.00019381	0	66.59881471	98.33123171
**3IPMDH_h**	0.806508612	amino acid synthesis	TRUE	0	0.897190548	5.64E-09	0.00019381	1	63.69446886	96.93378819
**5M_DASH_THFOR_c**	0.170753709	amino acid synthesis	TRUE	1	0.905223778	4.55E-09	0.00019381	1	26.42642791	79.96655404
**6PGDHNAD_h**	0	photosynthesis	FALSE	0	0.886498822	3.69E-08	0.00026262	64	18.31518129	100
**6PGDHNADP_h**	0	photosynthesis	FALSE	0	0.886991078	2.52E-08	0.00026849	26	16.45704297	100
**6PGL_h**	0	photosynthesis	FALSE	0	NA	4.8E-10	0.00019381	0		
**ACDOxRed_c**	4.610450529	amino acid synthesis	FALSE	1	0.942408966	1.26E-08	0.00026538	2	74.22647627	100
**ACoAC_h**	3.962009551	fatty acid synthesis	TRUE	1	0.949577575	5.23E-10	0.00019381	0	77.47300069	99.81742439
**ACoAS_h**	4.939271873	fatty acid synthesis	TRUE	1	0.948693118	1.55E-08	0.00021798	1	73.48567488	100
**ACPM_h**	3.962009551	fatty acid synthesis	TRUE	1	0.949652757	4.24E-10	0.00019381	0	81.17277425	100
**ADNK_c**	9.72E-15	nucleotide metabolism	FALSE	0	NA	5.16E-10	0.00019381	0		
**ADNN_c**	-9.72E-15	nucleotide metabolism	FALSE	0	NA	7.61E-09	0.00019381	1		
**ADPR_c**	0.003458813	nucleotide metabolism	TRUE	1	0.947910937	3.15E-09	0.00025905	0	96.82082821	100.0000002
**ADPRPPT_c**	-9.72E-15	nucleotide metabolism	FALSE	0	NA	5.16E-10	0.00019381	0		
**AGluK**	0.401851676	amino acid synthesis	TRUE	0	0.892189293	6.54E-10	0.00019381	0	23.2108314	100
**AGluPR_h**	0.401851676	amino acid synthesis	TRUE	0	0.885261553	6.69E-08	0.00019381	2	25.90458391	100
**AGNDA_h**	0.338621087	amino acid synthesis	TRUE	1	0.94132141	5.73E-10	0.00019381	0	47.42357964	86.2933466
**AGNDH_h**	0.238893923	amino acid synthesis	TRUE	0	0.877780403	6.44E-10	0.00019381	0	44.03616242	99.17037736
**AGPase_h**	3.022123708	starch synthesis	TRUE	1	0.949646468	2.35E-09	0.00019381	0	23.79611778	80.19596691
**AICARFT_h**	0.179247057	nucleotide metabolism	TRUE	1	0.94331558	4.42E-10	0.00019381	0	32.78987376	93.88205791
**AIRC_h**	0.010376237	nucleotide metabolism	TRUE	1	0.947721647	2.76E-09	0.00020983	0	96.78873564	100.0000002
**AlaTA_h**	0	amino acid synthesis	FALSE	0	NA	4.41E-08	0.00023626	10		
**AlaTA_m**	2.663204069	amino acid synthesis	FALSE	0	0.884986871	1.32E-10	0.00019381	0	0.713914307	46.48415241
**AlaTA_p**	-1.491466382	amino acid synthesis	FALSE	0	0.644940478	5.94E-09	0.00019381	0	1.82E-15	100
**ALS1_h**	2.005802107	amino acid synthesis	TRUE	1	0.949830036	2.19E-08	0.00019381	1	73.79077755	100
**ALS2_h**	0.456292823	amino acid synthesis	TRUE	0	0.89688981	1.34E-08	0.00019381	0	51.25673125	100
**ALS3_h**	1.549509284	amino acid synthesis	TRUE	0	0.888739154	8.3E-09	0.00019381	0	70.67813398	100
**AMPDA_c**	0.003942806	nucleotide metabolism	TRUE	1	0.951461698	4.47E-09	0.00022167	0	97.37400438	100.0000002
**AMPK_c**	0.347961016	nucleotide metabolism	TRUE	1	0.946211654	1.14E-08	0.00019381	0	2.106441777	100
**AMPK_h**	0	nucleotide metabolism	FALSE	0	NA	3.2E-08	0.00025905	3		
**AMT_m**	1.69775767	photorespiration	FALSE	1	0.926298495	8.22E-09	0.00019381	3	60.78814933	100
**ANTPRT_h**	0.085699644	amino acid synthesis	TRUE	1	0.948409062	5.47E-09	0.00019381	1	28.75312237	90.88614242
**ANTS_h**	0.085699644	amino acid synthesis	TRUE	1	0.948865877	6.17E-09	0.00019381	1	25.47914055	98.6058465
**AOrnAGluAcT_h**	0.401851676	amino acid synthesis	TRUE	0	0.892285503	3.72E-08	0.00019381	0	22.77297461	100
**AOrnTA_h**	0.401851676	amino acid synthesis	TRUE	0	0.885433866	4.61E-08	0.00019381	0	22.34082789	100
**APSR_h**	0.328821344	sulfur assimilation	FALSE	0	0.769711603	1.21E-08	0.0002031	8	1.78E-12	100.0000002
**Arginase_m**	0.014428511	amino acid synthesis	TRUE	0	0.893168667	8.28E-08	0.00019381	1	1.215024391	100
**ArgSCAL_h**	0.393792541	amino acid synthesis	TRUE	0	0.891041952	3.89E-08	0.00019381	0	25.91295243	100
**ArgSCAS_h**	0.393792541	amino acid synthesis	TRUE	0	0.867535306	9.54E-08	0.00021903	2	27.94767983	100
**Asnase_c**	0	amino acid synthesis	FALSE	0	NA	5.95E-10	0.00019381	0		
**AsnS_c**	0.358337253	amino acid synthesis	TRUE	1	0.933199159	3.51E-09	0.00019381	0	2.21470616	77.92746079
**Asp_DASH_SeADH_h**	6.458028394	amino acid synthesis	TRUE	1	0.948833574	5.56E-09	0.00020988	1	76.48122881	100
**AspAT_c**	0	amino acid synthesis	FALSE	0	0.892486194	5.56E-10	0.00019381	0	0.833936675	22.55287978
**AspAT_h**	7.052649624	amino acid synthesis	TRUE	1	0.948903809	2.06E-09	0.00019381	0	77.58972821	100
**AspAT_m**	0	amino acid synthesis	FALSE	0	NA	4.87E-10	0.00019381	0		
**AspAT_p**	0.871579473	amino acid synthesis	FALSE	0	0.579262892	8.12E-10	0.00019381	0	6.07E-16	10.40162365
**AspCT_h**	0.011205395	nucleotide metabolism	TRUE	1	0.949157168	2.89E-09	0.00026512	0	96.60160494	100
**AspK_h**	6.458028394	amino acid synthesis	TRUE	1	0.940812636	7.54E-09	0.00022256	0	79.28334189	100
**ATase_h**	0.010376237	nucleotide metabolism	TRUE	1	0.948479287	7.36E-09	0.00019584	0	97.06085657	100
**ATPase_h**	107.1428571	photosynthesis	TRUE	1	0.943521748	4.37E-09	0.00022427	0	97.54878627	100
**ATPCitL_c**	0.504474011	fatty acid synthesis	TRUE	0	0.881995955	5.23E-10	0.00019381	0	0.760928135	89.01765402
**ATPPRT_h**	0.16887082	amino acid synthesis	TRUE	0	0.897728939	5.65E-10	0.00019381	0	28.37797031	89.1378728
**ATPSL_h**	0.328821344	sulfur assimilation	FALSE	0	0.779207563	2.18E-08	0.0002493	36	2.03E-12	100.0000002
**BAATA1_h**	0.456292823	amino acid synthesis	TRUE	0	0.897130202	6.06E-09	0.00019381	0	48.86374856	100
**BAATA2_h**	0.806508612	amino acid synthesis	TRUE	1	0.949631358	1.6E-09	0.00019381	0	70.10104575	99.91601117
**BAATA3_h**	0.743000672	amino acid synthesis	TRUE	1	0.903962845	4.81E-08	0.00019381	0	56.11192262	100.0000001
**bAMY1_h**	0	starch degradation	FALSE	0	NA	2.22E-09	0.00019381	0		
**bAMY2_h**	0.013255864	starch degradation	FALSE	2	0.934986103	2.8E-09	0.00019381	0	0.161118445	66.10591348
**cACNDHA_c**	2.134753859	pyruvate metabolism	FALSE	0	0.897408819	2.49E-08	0.00027036	112	91.31057578	100
**cACNDHA_h**	0	pyruvate metabolism	FALSE	0	NA	2.26E-08	0.00022624	4		
**cACNDHA_m**	0	TCA	FALSE	1	0.930498499	1.59E-08	0.00019381	0	0	100
**cACNHA_c**	2.134753859	pyruvate metabolism	FALSE	1	0.930003478	3.04E-08	0.00027368	85	0	100
**cACNHA_h**	0	pyruvate metabolism	FALSE	0	NA	3.29E-08	0.00019682	8		
**cACNHA_m**	0	TCA	FALSE	0	0.897318223	1.89E-08	0.00027036	14	86.42394742	100
**CAT_h**	0.005188118	amino acid synthesis	TRUE	1	0.948517484	6.01E-09	0.00019381	1	1.064879874	13.71205756
**CAT_p**	0	photorespiration	FALSE	0	NA	6.11E-10	0.00019381	0		
**CBPS_h**	0.404997936	amino acid synthesis	TRUE	0	0.890806736	1.55E-08	0.00019381	0	28.38384576	100
**CDPR_c**	0.002050469	nucleotide metabolism	TRUE	1	0.942739394	6.73E-09	0.00026342	0	96.99435575	100.0000002
**CeS_c1**	1.848414034	cellulose synthesis	TRUE	1	0.933853741	1.8E-09	0.00019381	0	91.55907849	100
**CeS_c2**	1.848414034	cellulose synthesis	TRUE	1	0.936800648	3.29E-10	0.00019381	0	90.46639558	100
**CeS_c3**	0	cellulose synthesis	FALSE	0	NA	2.21E-08	0.00019381	1		
**CHRM_c**	0.57751501	amino acid synthesis	TRUE	1	0.9424527	5.73E-10	0.00019381	0	65.52030156	96.82595997
**CHRS_h**	0.663214654	shikimate pathway	TRUE	0	0.897661111	5.21E-10	0.00019381	0	66.875812	95.80667224
**CitS_c**	0	pyruvate metabolism	FALSE	0	NA	4.75E-10	0.00019381	0		
**CitS_h**	0	pyruvate metabolism	FALSE	0	NA	1.74E-08	0.00019381	4		

**CitS_m**	2.639227869	TCA	TRUE	0	0.8945403	3.42E-09	0.00024934	0	41.94992084	100
**cplx1_m**	8.630364043	oxidative phosphorylatio	FALSE	0	0.895157803	1.15E-08	0.00019381	2	33.9825201	100
**cplx2_m**	0.341392262	TCA	TRUE	1	0.949675762	4.19E-08	0.00019381	1	20.81242908	100.0000001
**cplx3_m**	8.982961701	oxidative phosphorylatio	TRUE	0	0.895189761	1.06E-08	0.0002076	0	33.89827618	100
**cplx4_m**	4.49148085	oxidative phosphorylatio	TRUE	0	0.895196282	2.38E-09	0.00019381	0	33.43247865	100
**cplx5_m**	22.10480659	oxidative phosphorylatio	TRUE	0	0.895199318	1.63E-08	0.00023146	0	38.61773709	100.0000001
**CTHL_h**	0.170753709	amino acid synthesis	TRUE	1	0.94881518	8.6E-09	0.00019381	1	25.27522691	86.89118602
**CTHS_h**	0.170753709	amino acid synthesis	TRUE	0	0.897809677	1.41E-08	0.00019381	1	20.98508201	100
**CTPP_c**	0.002050469	nucleotide metabolism	TRUE	1	0.947249804	2.83E-09	0.00025416	0	96.64380905	100.0000002
**CTPS_c**	0.004571926	nucleotide metabolism	TRUE	1	0.943622042	8.08E-09	0.00023308	1	96.99246218	100.0000002
**Cysase_c**	1.71E-16	amino acid synthesis	FALSE	0	NA	5.73E-10	0.00019381	0		
**CysS_c**	0.158067635	amino acid synthesis	TRUE	1	0.925218004	9.58E-09	0.00019381	0	12.51309288	100
**CysS_h**	0.170753709	amino acid synthesis	TRUE	0	0.889186533	4.5E-10	0.00019381	0	18.3908725	70.7945168
**CysS_m**	0	amino acid synthesis	FALSE	0	NA	7.66E-09	0.00025704	0		
**Cytb6f_h**	250	photosynthesis	TRUE	1	0.948547671	3.5E-09	0.00023642	1	98.02280678	100
**dADPK_c**	0.003458813	nucleotide metabolism	TRUE	1	0.948002089	5.93E-09	0.00022356	0	97.04075113	100.0000002
**DAHPS_h**	0.665141076	shikimate pathway	TRUE	1	0.948765711	5.65E-10	0.00019381	0	70.57909191	98.80846455
**DAPAT_h**	0.526223222	amino acid synthesis	TRUE	1	0.950160683	6.28E-10	0.00019381	0	56.55292593	95.19991527
**DAPDC_h**	0.526223222	amino acid synthesis	TRUE	1	0.950138584	5.15E-10	0.00019381	0	52.33021435	98.2843145
**DAPE_h**	0.526223222	amino acid synthesis	TRUE	1	0.950230888	5.15E-10	0.00019381	0	54.19608624	96.88925134
**DCAMPL1_h**	0.010376237	nucleotide metabolism	TRUE	1	0.947911309	5.5E-09	0.00026262	0	97.09959816	100
**DCAMPL2_h**	0.179247057	nucleotide metabolism	TRUE	1	0.947468034	4.7E-10	0.00019381	0	28.05306843	98.25191838
**DCAMPS_h**	0.179247057	nucleotide metabolism	TRUE	1	0.938337696	5.21E-09	0.00019381	1	33.5272317	99.24350428
**dCDPK_c**	0.002050469	nucleotide metabolism	TRUE	1	0.946947711	1.74E-09	0.00026104	0	96.82003935	100.0000002
**dGDPK_c**	0.001886649	nucleotide metabolism	TRUE	1	0.947141172	3.75E-09	0.00025748	0	97.16838443	100.0000002
**DHADA1_h**	0.456292823	amino acid synthesis	TRUE	1	0.950485277	9.2E-09	0.00019381	0	48.77752376	100
**DHADA2_h**	1.549509284	amino acid synthesis	TRUE	1	0.945492422	1.94E-08	0.00019381	1	71.85199889	100
**DHDRNAD_h**	0.526223222	amino acid synthesis	FALSE	1	0.911459722	1.81E-08	0.00026434	28	0	100
**DHDRNADP_h**	0	amino acid synthesis	FALSE	0	0.894162132	1.74E-08	0.00025115	39	47.04626022	100
**DHDS_h**	0.526223222	amino acid synthesis	TRUE	1	0.950221605	4.59E-09	0.00019381	1	52.02827863	96.27020961
**DHFR_c**	0.003519262	THF recycling	TRUE	1	0.948484899	2.79E-09	0.00026684	0	96.56610176	100.0000002
**DHLAcT_m**	2.639227869	TCA	TRUE	0	0.894575794	2.93E-09	0.00019381	3	40.99312114	100
**DHLDH1_m**	1.69775767	photorespiration	FALSE	1	0.926245436	7.23E-09	0.00020872	1	61.78705407	100
**DHLDH2_m**	2.639227869	TCA	TRUE	0	0.887202956	8.93E-09	0.00019381	0	40.24480196	100
**DHLST_m**	0	TCA	FALSE	0	NA	2.16E-08	0.00021351	9		
**DHOase_h**	0.011205395	nucleotide metabolism	TRUE	1	0.948312176	3.52E-09	0.00022186	0	96.61760593	100
**DHODH_m**	0.011205395	nucleotide metabolism	TRUE	1	0.94907616	2.73E-09	0.00026747	0	96.69127402	100
**DHQDH_h**	0.665141076	shikimate pathway	TRUE	0	0.898136992	4.64E-09	0.00019381	1	68.5477072	100
**DHQS_h**	0.665141076	shikimate pathway	TRUE	1	0.948915667	1.25E-09	0.00019381	1	65.17362262	100
**DPE11_h**	0	starch degradation	FALSE	0	NA	2.96E-08	0.00019381	5		
**DPE12_h**	0	starch degradation	FALSE	0	NA	2.09E-09	0.00019381	0		
**DPE13_h**	0	starch degradation	FALSE	0	NA	9.69E-09	0.00023156	0		
**DPE2_c**	0	starch degradation	FALSE	0	NA	3.26E-09	0.00019381	0		
**dTDPK_c**	0.003519262	nucleotide metabolism	TRUE	1	0.948701719	2.31E-09	0.00027309	0	96.74987353	100.0000002
**dTMPK_c**	0.003519262	nucleotide metabolism	TRUE	1	0.94844726	6.64E-09	0.00021082	0	96.53154506	100.0000002
**dTMPS_c**	0.003519262	nucleotide metabolism	TRUE	1	0.948436075	6.81E-09	0.00025963	0	96.6318497	100.0000002
**dUDPK_c**	0.003519262	nucleotide metabolism	TRUE	1	0.940235704	3.41E-09	0.00019381	0	96.73735653	100.0000002
**dUTPP_c**	0.003519262	nucleotide metabolism	TRUE	1	0.947971935	7.15E-09	0.00026849	0	97.03833159	100.0000002
**Enol_c**	-9.388206824	gluconeogenesis	FALSE	1	0.949713928	1.56E-08	0.00019381	0	9.359484273	100
**Enol_h**	8.34823255	glycolysis	FALSE	0	0.887619213	1.86E-08	0.00023503	23	6.568485012	100
**EPSPS_h**	0.663214654	shikimate pathway	TRUE	1	0.936194572	7.73E-10	0.00019381	0	67.06825613	92.55054399
**F26BPPh_c**	0	sucrose synthesis	FALSE	0	NA	5.04E-10	0.00019381	0		
**F6PK1_c**	0	glycolysis	FALSE	0	NA	5.14E-10	0.00019381	0		
**F6PK1_h**	0	glycolysis	FALSE	0	NA	2.07E-09	0.00019381	0		
**F6PK2_c**	0	sucrose synthesis	FALSE	0	NA	5.41E-10	0.00019381	0		
**FBPA_c**	4.169265067	sucrose synthesis	FALSE	0	0.853446394	2.82E-08	0.00022256	6	23.96793448	100
**FBPA_h**	70.72284906	photosynthesis	FALSE	0	0.884120197	2.44E-08	0.00026826	178	93.91461563	100
**FBPase_c**	0	sucrose synthesis	FALSE	0	0.853546662	8.08E-09	0.00025113	2	13.63331968	100
**FBPase_h**	70.72284906	photosynthesis	FALSE	0	0.881780152	2.82E-08	0.00027368	134	69.5946744	100
**Fd_DASH_NADPR_h**	210.4177111	photosynthesis	FALSE	1	0.908566801	1.88E-08	0.00026573	27	100	100
**FGAMCL_h**	0.010376237	nucleotide metabolism	TRUE	1	0.947721647	5.5E-09	0.00022995	0	96.30398912	100.0000002
**FGAMS_h**	0.010376237	nucleotide metabolism	TRUE	1	0.948494986	2.53E-09	0.00024934	0	97.0600881	100
**ForDH_h**	0.189623294	THF recycling	TRUE	1	0.949491336	5.03E-10	0.00019381	0	33.98977715	97.83476448
**ForTHFL_h**	0.189623294	THF recycling	TRUE	1	0.948925774	4.78E-10	0.00019381	0	30.51986945	98.31004487
**FrcFD_c**	0.093866011	sucrose degradation	FALSE	1	0.949629376	5.73E-10	0.00019381	0	0.310377022	10.5811691
**FrcK_c**	0	sucrose degradation	FALSE	0	NA	1.06E-09	0.00019381	0		
**FTK_h**	34.18293322	photosynthesis	TRUE	1	0.939375282	6.12E-09	0.00023024	0	95.44222057	100
**FumHA_c**	0.583415835	nucleotide metabolism	TRUE	0	0.885071494	3.15E-08	0.00019381	2	32.85559222	100
**FumHA_m**	0	TCA	FALSE	0	NA	2.1E-08	0.00019381	0		
**G6PDH_h**	0	photosynthesis	FALSE	0	NA	4.48E-10	0.00019381	0		
**GABATA1_m**	0	glutamate degradation	FALSE	0	0.837863263	5.08E-10	0.00019381	0	0.100967125	4.052662144
**GABATA2_m**	0	glutamate degradation	FALSE	0	0.487898721	6.24E-10	0.00019381	0	0	3.220042893
**GAPDH1_h**	200.7969604	photosynthesis	FALSE	0	0.863695904	3.06E-08	0.00026849	177	100	100
**GAPDH2_c**	18.72120015	gluconeogenesis	FALSE	0	0.899936006	1.44E-08	0.00024934	30	56.49421625	100
**GAPDH2_h**	-1.805701541	glycolysis	FALSE	1	0.923698287	4.61E-08	0.00026849	49	37.40648781	100
**GAPDH3_c**	0	glycolysis	FALSE	0	NA	7.78E-09	0.00019381	1		
**GARFT_h**	0.010376237	nucleotide metabolism	TRUE	1	0.948444254	4.88E-09	0.0002561	0	96.77114602	100.0000002
**GCAO_h**	0.010376237	amino acid synthesis	TRUE	1	0.948505005	5.87E-10	0.00019381	0	1.041355417	34.78395947
**GCAO_p**	0	photorespiration	FALSE	0	NA	6.26E-10	0.00019381	0		
**GCEADH_h**	0	amino acid synthesis	FALSE	0	NA	5.14E-10	0.00019381	0		
**GCEADH_p**	0	photorespiration	FALSE	0	NA	5.11E-10	0.00019381	0		

**GCEAK_h**	0	photorespiration	FALSE	0	NA	7.32E-09	0.00019381	0		
**GDPK_c**	0.002056158	nucleotide metabolism	TRUE	1	0.951461941	2.47E-09	0.00021994	0	96.7156217	100.0000002
**GDPK_h**	0.179247057	nucleotide metabolism	TRUE	0	0.899177761	5.76E-10	0.00019381	0	30.72857657	69.83793586
**GDPR_c**	0.001886649	nucleotide metabolism	TRUE	1	0.948164947	5.4E-09	0.00023233	0	96.6587144	100.0000002
**GGAT_h**	0.010376237	amino acid synthesis	FALSE	1	0.948498782	2.67E-09	0.00019381	0	1.044405711	36.80994976
**GGAT_p**	0	photorespiration, glycine	FALSE	0	NA	5.12E-10	0.00019381	0		
**Glnase_c**	0	amino acid synthesis	FALSE	0	NA	4.77E-10	0.00019381	0		
**GlnS_c**	0.627258863	amino acid synthesis	TRUE	1	0.950953594	6.24E-10	0.00019381	0	2.077413939	30.08927982
**GlnS_h**	11.68006292	amino acid synthesis	TRUE	0	0.890484598	3.08E-08	0.0002451	22	67.12143475	100
**GlnS_m**	0	amino acid synthesis	FALSE	0	NA	5.23E-09	0.00019381	1		
**GluDC_c**	0.000477542	glutamate degradation	TRUE	1	0.949771101	4.46E-08	0.00025483	4	14.85265562	100.0000002
**GluDH1NAD_m**	2.043323103	amino acid synthesis	FALSE	1	0.915648743	3.56E-09	0.00019381	1	0.270134634	15.58341227
**GluDH1NADP_m**	0	amino acid synthesis	FALSE	0	0.709117537	6.71E-10	0.00019381	0	0	22.01732521
**GluDH2NAD_m**	0	glutamate degradation	FALSE	0	0.722611991	5.25E-10	0.00019381	0	0	15.69332027
**GluDH3NADP_c**	-1.773913029	amino acid synthesis	FALSE	0	0.893447261	3.81E-09	0.00019381	1	2.858645492	100
**GluK_c**	0	amino acid synthesis	FALSE	0	0.361938265	2.44E-09	0.00019381	0	0	100
**GluK_h**	0	amino acid synthesis	FALSE	0	NA	5.06E-08	0.00023642	21		
**GluK_m**	0.534365613	amino acid synthesis	FALSE	0	0.861229604	1.17E-08	0.00019381	1	0.718072958	100
**GluSeADA_c**	0	amino acid synthesis	FALSE	0	0.521195255	5.21E-10	0.00019381	0	0	78.65245648
**GluSeADA_h**	0	amino acid synthesis	FALSE	0	NA	3.43E-08	0.00025675	23		
**GluSeADA_m**	0.548794124	amino acid synthesis	TRUE	0	0.886579572	5.58E-09	0.00019381	2	0.839853283	100
**GluSeADH_c**	0	amino acid synthesis	FALSE	0	0.521195255	7.11E-10	0.00019381	0	0	85.53495629
**GluSeADH_h**	0	amino acid synthesis	FALSE	0	NA	5.69E-08	0.00025675	24		
**GluSeADH_m**	0.534365613	amino acid synthesis	FALSE	0	0.861261402	5.38E-08	0.00019381	1	0.773996305	100
**GluSFd_h**	10.99974205	amino acid synthesis	FALSE	1	0.947423995	2.23E-08	0.00026512	137	51.62788592	100
**GluSNAD_h**	0	amino acid synthesis	FALSE	0	NA	1.46E-08	0.00019381	0	0	0
**GluSNADP_h**	0	amino acid synthesis	FALSE	1	0.946162942	3.02E-08	0.00027368	133	30.8556023	100
**GlyDH_m**	1.69775767	photorespiration	FALSE	0	0.894323883	1.63E-08	0.00026512	2	61.98376661	100
**GlyHMT_c**	-0.174272971	amino acid synthesis	TRUE	1	0.950487315	5.36E-09	0.00019381	1	26.05231085	76.95538996
**GlyHMT_m**	1.69775767	photorespiration	FALSE	0	0.894353144	8.39E-09	0.00025982	2	59.1378382	100
**GMPK_c**	0.003942806	nucleotide metabolism	TRUE	1	0.951462249	7.02E-09	0.00019381	0	96.86780604	100.0000002
**GMPS_c**	0.003942806	nucleotide metabolism	TRUE	1	0.944959951	5.64E-09	0.00023568	1	96.93995341	100
**GPAT_h**	0.57751501	amino acid synthesis	TRUE	1	0.949154008	1.37E-09	0.00019381	0	64.02892411	100
**GSSGR_h**	0.328821344	sulfur assimilation	FALSE	0	0.802746658	2.7E-08	0.00024934	72	1.96E-12	100.0000002
**HCO3DHA_c**	9.388206824	photosynthesis	FALSE	1	0.949750386	1.56E-09	0.00019381	0	21.98928633	100
**HCO3DHA_h**	4.367007487	photosynthesis	TRUE	0	0.884590577	1.43E-09	0.00019381	0	79.56373049	100
**HCysMT_c**	0	amino acid synthesis	FALSE	0	NA	1.57E-08	0.00019381	1		
**HEXK_c**	0	starch degradation, sucr	FALSE	0	NA	5.5E-10	0.00019381	0		
**HisalPDH_h**	0.16887082	amino acid synthesis	TRUE	1	0.949020127	4.79E-10	0.00019381	0	30.68232795	91.16058482
**HisolPDH_h**	0.16887082	amino acid synthesis	TRUE	1	0.948995718	4.92E-09	0.00019381	1	30.42526019	91.45473596
**HisolPP_h**	0.16887082	amino acid synthesis	TRUE	0	0.897633045	4.97E-09	0.00019381	1	28.77797236	86.83984078
**HisolPTA_h**	0.16887082	amino acid synthesis	TRUE	1	0.930307205	8.67E-09	0.00019381	1	30.11282252	88.88325072
**HSerDHNAD_h**	5.931805173	amino acid synthesis	FALSE	0	0.866395526	1.59E-08	0.00027368	69	46.22997082	100
**HSerDHNADP_h**	0	amino acid synthesis	FALSE	0	0.885859302	2.65E-08	0.00026512	109	88.91135585	100
**HSerK_h**	5.931805173	amino acid synthesis	TRUE	1	0.904169945	7.6E-09	0.00019381	0	74.68357687	100
**iCitDHNAD_c**	0	pyruvate metabolism	FALSE	0	0.815971618	3.25E-08	0.00025982	20	0.527587676	100
**iCitDHNAD_m**	0	TCA	FALSE	0	0.868660005	3.11E-09	0.00019381	0	0	31.68552535
**iCitDHNADP_c**	1.788347483	pyruvate metabolism	FALSE	0	0.856980367	2.03E-08	0.00026167	96	91.24037968	100
**iCitDHNADP_h**	0	pyruvate metabolism	FALSE	0	NA	2.65E-08	0.00019381	5		
**iCitDHNADP_m**	0	pyruvate metabolism	FALSE	0	0.371339795	8.49E-10	0.00019381	0	0	38.06514015
**iCitL_c**	0.346406376	pyruvate metabolism	FALSE	0	0.815661816	1.02E-08	0.00022995	2	0	100
**IGPDA_h**	0.16887082	amino acid synthesis	TRUE	1	0.948922584	5.67E-10	0.00019381	0	32.37553998	95.15880761
**IGPS_h**	0.16887082	amino acid synthesis	TRUE	1	0.949009733	5.73E-10	0.00019381	0	30.19587263	93.0951857
**Im_NO3**	9.36310494	transport	FALSE	0	0.877485875	1.07E-08	0.00026673	8	87.52535929	100
**Im_Pi**	0.064744896	transport	TRUE	1	0.948625395	3.48E-09	0.00019381	0	96.72222173	100
**Im_SO4**	0.328821344	transport	FALSE	0	0.808366172	2.17E-08	0.00026573	43	2.16E-12	100.0000002
**IMPCH_h**	0.179247057	nucleotide metabolism	TRUE	1	0.947451469	5.21E-10	0.00019381	0	33.10863695	90.03062468
**IMPDH_c**	0.003942806	nucleotide metabolism	TRUE	1	0.951461698	3.97E-09	0.00024318	0	96.63118318	100
**IndGPL_h**	0.085699644	amino acid synthesis	TRUE	1	0.94421273	4.75E-10	0.00019381	0	29.24594074	97.53416846
**IndGPS_h**	0.085699644	amino acid synthesis	TRUE	1	0.937227622	4.35E-09	0.00019381	1	28.28416556	97.84997192
**IPODC_h**	0.806508612	amino acid synthesis	TRUE	0	0.897182174	4.81E-10	0.00019381	0	65.36797528	98.06169056
**KARI1_h**	0.456292823	amino acid synthesis	TRUE	0	0.896898774	3.56E-09	0.00019381	0	47.18534954	100
**KARI2_h**	0.456292823	amino acid synthesis	TRUE	1	0.950474982	2.33E-09	0.00019381	0	50.58787538	100
**KARI3_h**	1.549509284	amino acid synthesis	TRUE	1	0.949587433	2.22E-08	0.00019381	0	71.33458674	100
**KARI4_h**	1.549509284	amino acid synthesis	TRUE	1	0.930801672	3.35E-08	0.00019381	2	73.40770958	100
**KGDH_m**	0	TCA	FALSE	0	NA	1.83E-08	0.0001985	11		
**MalDH1_c**	10.02646333	gluconeogenesis	FALSE	0	0.88507059	1.27E-08	0.00023551	5	14.64494031	100
**MalDH1_h**	7.052649624	pyruvate metabolism	TRUE	2	0.948811077	4.14E-08	0.00024054	7	8.883340189	100
**MalDH1_m**	-16.40833683	TCA	FALSE	0	0.890273145	7.14E-09	0.00021563	0	11.13667205	100
**MalDH1_p**	0	photorespiration	FALSE	0	NA	3.68E-10	0.00019381	0		
**MalDH2NADP_c**	0	pyruvate metabolism	FALSE	0	NA	4.39E-08	0.00019381	0		
**MalDH2NADP_h**	0	pyruvate metabolism	FALSE	0	NA	1.4E-09	0.00020306	0		
**MalDH3_c**	0	pyruvate metabolism	FALSE	0	0.693416174	4.48E-10	0.00019381	0	0	36.21110735
**MalDH3_h**	0	pyruvate metabolism	FALSE	1	0.91555783	1.3E-08	0.00022256	1	0	100
**MalDH3_m**	0	pyruvate metabolism	FALSE	0	0.843300993	2.27E-08	0.00021716	28	0	100
**MalS_c**	0.346406376	pyruvate metabolism	FALSE	0	0.815654481	1.33E-08	0.00019381	10	0	100
**MetAdT_h**	9.19E-15	amino acid synthesis	FALSE	0	NA	5.37E-09	0.00019381	1		
**MetS_c**	0.170753709	amino acid synthesis	TRUE	1	0.950474901	4.95E-09	0.00019381	1	26.65820434	96.0960361
**NGAM_c**	0	maintenance	FALSE	0	NA	5.48E-10	0.00019381	0		
**NGAM_h**	0	maintenance	FALSE	0	NA	4.23E-08	0.00019381	0		
**NGAM_m**	0	maintenance	FALSE	0	NA	2.94E-08	0.00023245	10		

**NMPK_c**	0.011205395	nucleotide metabolism	TRUE	1	0.949132403	4.2E-09	0.00019381	0	96.29421754	100.0000002
**NO2R_h**	9.36310494	nitrate assimilation	FALSE	0	0.877432541	7.9E-09	0.00027309	9	86.21001724	100
**NO3R1_c**	9.36310494	nitrate assimilation	FALSE	0	0.884641568	1.16E-08	0.00019381	6	7.572721072	100
**NO3R2_c**	0	nitrate assimilation	FALSE	0	0.864172548	3.34E-08	0.00019484	13	1.585743906	100
**OMPDC_c**	0.011205395	nucleotide metabolism	TRUE	1	0.949181531	3.07E-09	0.0002237	0	96.58064652	100.0000002
**OrnAT_m**	0.014428511	amino acid synthesis	TRUE	0	0.893145034	3.48E-08	0.00023297	0	1.043088743	87.12727463
**OrnCMT_h**	0.393792541	amino acid synthesis	TRUE	0	0.891132433	5.15E-10	0.00019381	0	22.96874986	91.59733257
**OROPRT_c**	0.011205395	nucleotide metabolism	TRUE	1	0.949047694	5.05E-09	0.00026982	0	97.29419003	100
**P5CDHNAD_h**	0	proline degradation	FALSE	0	NA	1.22E-08	0.00021716	0		
**P5CDHNAD_m**	0	proline degradation	FALSE	0	NA	4.47E-09	0.00019381	0		
**P5CDHNADP_m**	0	proline degradation	FALSE	0	NA	4.62E-08	0.00019381	11		
**P5CRNAD_c**	23.82819817	amino acid synthesis	TRUE	0	0.89275953	2.29E-08	0.00019491	28	46.10033028	100
**PAICARPI_h**	0.16887082	amino acid synthesis	TRUE	1	0.948807056	5.73E-10	0.00019381	0	32.64321027	85.04103977
**PEPC1_c**	0	gluconeogenesis	FALSE	0	NA	1.34E-09	0.00019381	0		
**PEPC2_c**	-9.388206824	gluconeogenesis	FALSE	1	0.949665977	4.65E-10	0.00019788	0	18.95913364	100
**PGADH_h**	0.256453353	amino acid synthesis	TRUE	0	0.880454338	4.15E-09	0.00019381	0	7.11621646	100
**PGAK_c**	-18.72120015	gluconeogenesis	FALSE	0	0.899736625	2.05E-08	0.00027368	46	60.40340408	100
**PGAK_h**	202.602662	photosynthesis	FALSE	1	0.947434825	4.03E-09	0.00023551	0	91.90663957	100
**PGAM_c**	-9.388206824	gluconeogenesis	FALSE	1	0.949681679	6.13E-09	0.00019381	1	7.296046433	49.81101687
**PGAM_h**	8.34823255	glycolysis	FALSE	0	0.887647347	5.68E-09	0.00019381	1	5.654245647	85.04625757
**PGI_c**	3.952345589	sucrose synthesis	FALSE	0	0.853752909	3.52E-08	0.00025115	25	21.55244098	100
**PGI_h**	3.022123708	starch synthesis	FALSE	0	0.857752628	3.63E-08	0.00026512	25	19.47242927	100
**PGM_c**	3.949412778	sucrose synthesis	FALSE	1	0.949751841	3.06E-09	0.00019381	0	16.38192908	100
**PGM_h**	3.022123708	starch synthesis	TRUE	1	0.947200924	1.62E-08	0.00019381	0	26.26312651	100
**PGP_h**	0.010376237	photorespiration	TRUE	1	0.948516717	5.57E-09	0.00019381	0	0.802859565	100
**PPIase_c**	8.495682561	pyrophosphate recycling	FALSE	1	0.948695739	1.26E-08	0.00020236	6	7.725343469	100
**PPIase_h**	2.990165839	pyrophosphate recycling	TRUE	1	0.947377397	2.44E-08	0.00019381	1	26.54241335	100
**PPIF6PK_c**	-4.169265067	pyrophosphate recycling	FALSE	0	0.783060417	2.13E-08	0.00023626	2	0	54.46395555
**PRAGlyL_h**	0.010376237	nucleotide metabolism	TRUE	1	0.948538607	5.26E-09	0.00025982	0	97.0896518	100
**PrAMPCy_h**	0.16887082	amino acid synthesis	TRUE	1	0.949017802	5.22E-10	0.00019381	0	29.45288929	82.60588248
**PRANTI_h**	0.085699644	amino acid synthesis	TRUE	1	0.948647479	4.77E-09	0.00019381	1	24.96274301	89.95103176
**PrATPDP_h**	0.16887082	amino acid synthesis	TRUE	0	0.897516152	5.65E-10	0.00019381	0	32.1012975	97.59943896
**ProDHNAD_m**	22.74503843	proline degradation	FALSE	0	0.858764105	1.92E-08	0.0001985	6	51.69060871	100
**ProDHNADP_m**	0.534365613	proline degradation	FALSE	0	0.852177841	2.15E-08	0.00026434	47	1.24596402	100
**PSerAT_h**	0.256453353	amino acid synthesis	TRUE	1	0.948899559	7.42E-09	0.00019381	0	6.268207469	96.18678472
**PSerP_h**	0.256453353	amino acid synthesis	TRUE	1	0.948780218	5.74E-10	0.00019381	0	6.340263498	60.51500723
**PSI_h**	500	photosynthesis	TRUE	1	0.947600005	7.06E-09	0.00027368	0	98.47747863	100
**PSII_h**	125	photosynthesis	TRUE	1	0.943519845	4.92E-09	0.00022509	1	97.61033695	100
**PyrDH1_m**	2.639227869	TCA	TRUE	0	0.894576929	5.14E-09	0.00019381	0	39.44267581	100
**PyrDH2_m**	2.639227869	TCA	TRUE	0	0.895163907	9.4E-09	0.00019381	0	41.72473632	100
**PyrK_c**	0	glycolysis	FALSE	0	NA	4.13E-08	0.00019381	3		
**PyrK_h**	0.892215673	glycolysis	FALSE	0	0.832158872	2.46E-08	0.00022518	13	6.686917086	100
**PyrPiDK_h**	-6.127661148	pyruvate metabolism	FALSE	1	0.908925841	8.9E-09	0.00027368	21	80.94452109	100.0000001
**R5PDPK_h**	0.276152096	PRPP synthesis	TRUE	1	0.948874813	4.02E-09	0.00019381	0	10.77639078	78.74017466
**R5PI_h**	33.24164004	photosynthesis	TRUE	1	0.942690349	4.96E-09	0.00025704	0	94.30406199	100
**RBC_h**	100.9319892	photosynthesis	TRUE	2	0.938173083	4.63E-09	0.00024589	0	95.34107326	100
**RBO_h**	0.010376237	photorespiration	TRUE	0	0.885083614	1.53E-09	0.00019381	0	0.613910882	99.99999999
**RibK_h**	-9.72E-15	nucleotide metabolism	FALSE	0	NA	5.66E-10	0.00019381	0		
**Ru5PE_h**	67.70072535	photosynthesis	TRUE	1	0.945651806	2.05E-09	0.00019381	0	95.27689499	100
**Ru5PK_h**	100.9423654	photosynthesis	TRUE	1	0.935398774	6.01E-09	0.00025115	1	95.90274319	100
**S6PPh_c**	0.216919478	sucrose synthesis	TRUE	1	0.949048264	5.19E-10	0.00019381	0	0.610464968	49.16854196
**S6PS_c**	0.216919478	sucrose synthesis	TRUE	1	0.948997824	5.21E-10	0.00019381	0	0.418088424	8.27691099
**SADH_h**	0.665141076	shikimate pathway	TRUE	0	0.890379706	7.02E-10	0.00019381	0	65.87602709	99.45374948
**SAICARS_h**	0.010376237	nucleotide metabolism	TRUE	1	0.940075024	5.36E-09	0.00026722	0	96.69083713	100
**SAK_h**	0.663214654	shikimate pathway	TRUE	1	0.949064919	5.08E-10	0.00019381	0	66.02402177	99.02166081
**SBPA_h**	0	photosynthesis	FALSE	1	0.943609981	2.08E-08	0.00025865	37	72.81851014	100
**SBPase_h**	0	photosynthesis	FALSE	2	0.951942949	1.17E-08	0.00026512	25	83.80840965	100
**SCACoAL_m**	0	TCA	FALSE	0	NA	2.1E-08	0.00021763	6		
**SCASeADH_m**	0	glutamate degradation	FALSE	0	NA	1.22E-08	0.00019381	0		
**SerAcT_c**	0.158067635	amino acid synthesis	TRUE	1	0.949586935	2.56E-09	0.00019381	0	15.10491195	100
**SerAcT_h**	0.170753709	amino acid synthesis	TRUE	1	0.948863303	4.5E-09	0.00019381	0	14.83331143	100
**SerAcT_m**	0	amino acid synthesis	FALSE	0	NA	1.25E-08	0.0002063	1		
**SerRM_c**	0.616169996	serine racemization	FALSE	0	0.859115415	9.53E-09	0.00021626	0	14.98878317	100
**SGAT_h**	0	amino acid synthesis	FALSE	0	NA	7.26E-09	0.00019381	1		
**SGAT_p**	0	photorespiration, glycine	FALSE	0	NA	4.8E-09	0.00019381	1		
**SO3R_h**	0.328821344	sulfur assimilation	FALSE	0	0.865916277	1.68E-08	0.00023229	22	3.66E-13	100.0000002
**STK_h**	33.51779214	photosynthesis	TRUE	1	0.948852456	1.49E-09	0.00019846	0	94.05421507	100
**StP1_h**	0	starch degradation	FALSE	0	NA	7.05E-10	0.00019381	0		
**StP21_c**	0	starch degradation	FALSE	0	NA	1.75E-08	0.00019381	0		
**StS_h1**	1.49780599	starch synthesis	TRUE	1	0.949571307	2.19E-09	0.00019381	0	30.00914015	99.7763762
**StS_h2**	1.511061854	starch synthesis	TRUE	1	0.949641871	2.28E-09	0.00019381	0	12.93516628	48.90140005
**StS_h3**	0.013255864	starch synthesis	TRUE	1	0.948926398	2.26E-09	0.00019381	0	0.183791522	36.53773679
**SucS_c**	-0.032732421	sucrose degradation	FALSE	1	0.94987155	5.21E-10	0.00019381	0	0.10046609	1.754687098
**TA_h**	-33.51779214	photosynthesis	FALSE	0	0.891210147	2.06E-08	0.00025115	81	17.34553585	100
**ThrA_c**	4.610450529	amino acid synthesis	FALSE	1	0.942378073	4.98E-09	0.00021684	3	73.36002649	100
**ThrDA_h**	0.456292823	amino acid synthesis	TRUE	1	0.950347196	4.72E-10	0.00019381	0	49.96665658	98.83257736
**ThrS_h**	5.761051463	amino acid synthesis	TRUE	1	0.910290458	1.07E-08	0.00026573	6	74.1596875	100
**TPI_c**	4.169265067	sucrose synthesis	FALSE	0	0.850275574	4.12E-09	0.00019381	0	0	100
**TPI_h**	70.72284906	photosynthesis	FALSE	2	0.94880565	1.76E-08	0.00026476	40	49.88220849	100
**TPP_c**	0.002932811	trehalose synthesis	TRUE	0	0.885666818	2.62E-09	0.00019381	0	1.159762953	10.30632557
**TPS_c**	0.002932811	trehalose synthesis	TRUE	0	0.889600536	2.62E-09	0.00019381	0	1.064540562	15.35465346

Tr_AAC	21.57044098	transport	FALSE	0	0.883288412	1.69E-08	0.00020691	9	40.39951787	100.0000001
Tr_AC1	4.768518164	transport	TRUE	1	0.948669892	4.98E-09	0.00019381	1	72.03018367	100
Tr_AC2	0	transport	FALSE	0	NA	1.1E-08	0.00020012	3		
Tr_Ala1	1.491466382	transport	FALSE	0	0.644818919	2.89E-10	0.00019381	0	1.83E-15	65.29648492
Tr_Ala2	0	transport	FALSE	0	NA	2.87E-08	0.00020236	2		
Tr_Arg1	0	transport	FALSE	0	NA	5.14E-10	0.00019381	0		
Tr_Arg2	0.393792541	transport	TRUE	0	0.891206366	9.08E-09	0.00019381	0	23.82906543	99.99999999
Tr_Asn1	0	transport	FALSE	0	NA	3.91E-09	0.00019381	1		
Tr_Asn2	0	transport	FALSE	0	NA	4.27E-09	0.00019381	1		
Tr_Asn3	0	transport	FALSE	0	NA	5.29E-10	0.00019381	0		
Tr_Asp	-0.871579473	transport	FALSE	0	0.667807187	5.13E-10	0.00019381	0	0	7.271482658
Tr_BAC1	0	transport	FALSE	0	NA	4.64E-10	0.00019381	0	0	0
Tr_BAC2	0	transport	FALSE	0	NA	4.58E-10	0.00019381	0	0	0
Tr_BAT11	-2.663204069	transport	FALSE	0	0.88936193	2.15E-09	0.00019381	0	0.739477688	100
Tr_BAT12	0.014428511	transport	FALSE	0	0.89316208	7.36E-09	0.00019381	1	0.008616452	3.159155579
Tr_BAT13	1.139818068	transport	FALSE	0	0.859714944	9.04E-09	0.00019381	0	2.152257159	100
Tr_BAT14	0	transport	FALSE	0	NA	4.25E-09	0.00019381	1	0	0
Tr_BAT15	0	transport	FALSE	0	NA	2.2E-08	0.00025675	3		
Tr_BT11	0	transport	FALSE	0	0.876458715	1.55E-08	0.00026516	63	43.73953421	100.0000002
Tr_BT12	0.010376237	transport	FALSE	0	0.869840067	2.02E-08	0.00027368	208	100	100.0000002
Tr_BT13	0	transport	FALSE	0	NA	1.66E-08	0.00019381	1		
Tr_CO2h	101.4972476	transport	FALSE	1	0.951321879	5.3E-09	0.00026573	1	96.19372559	100
Tr_CO2m	-4.33698554	transport	TRUE	0	0.895540459	6.18E-09	0.00019381	1	46.69435269	100
Tr_Cys	0	transport	FALSE	0	NA	6.26E-10	0.00019381	0		
Tr_DHO1	0.011205395	transport	TRUE	1	0.946183045	3.86E-09	0.0002536	0	96.96258441	100
Tr_DHO2	0.011205395	transport	TRUE	1	0.950346322	2.47E-09	0.00019381	0	96.42391364	100
Tr_DIC1	0	transport	FALSE	0	0.454632447	6.21E-10	0.00019381	0	0	3.008226295
Tr_DIC2	0	transport	FALSE	0	0.668984296	1.66E-09	0.00019381	0	5.91E-16	0.877396589
Tr_DIT1	0	transport	FALSE	0	NA	4.84E-08	0.00023626	35		
Tr_DIT2	7.052649624	transport	TRUE	0	0.765274142	4.78E-08	0.00021676	16	52.51932716	100
Tr_DTC1A	-0.605452455	transport	FALSE	0	0.708257123	5.64E-10	0.00019381	0	0	7.678244231
Tr_DTC1B	0	transport	FALSE	0	0.883165489	5.91E-10	0.00019381	0	0.290599102	5.057022778
Tr_DTC1C	0	transport	FALSE	0	0.356385868	5.56E-09	0.00019381	1	0	2.451471727
Tr_DTC2A	0	transport	FALSE	0	0.84519442	6.64E-09	0.00025935	0	16.33677539	100
Tr_DTC2B	-16.14929075	transport	FALSE	0	0.795869478	1.42E-08	0.00020304	9	0	17.94217128
Tr_DTC2C	0	transport	FALSE	0	0.897598027	3.55E-08	0.00019381	8	1.031068208	100
Tr_DTC3A	2.898273948	transport	FALSE	0	0.890269124	3.08E-10	0.00019381	0	15.12747545	100
Tr_DTC3B	16.14929075	transport	FALSE	0	0.894543222	2.39E-08	0.00027368	48	1.88022065	100
Tr_DTC3C	0	transport	FALSE	0	0.837161724	6.7E-09	0.00019381	1	0	30.09073227
Tr_DTC4A	0.346406376	transport	FALSE	0	0.778455834	6.64E-10	0.00019381	0	2.68E-15	8.069203498
Tr_DTC4B	0	transport	FALSE	0	0.673562408	6.34E-09	0.00019381	1	0	1.506051745
Tr_DTC4C	0	transport	FALSE	0	0.31832641	6.06E-09	0.00019381	1	0	1.007424029
Tr_Fum	0.583415835	transport	TRUE	0	0.891301656	6.16E-09	0.00019381	0	34.06609381	100
Tr_GCA1	0	transport	FALSE	0	NA	6.72E-10	0.00019381	0		
Tr_GCA2	0	transport	FALSE	0	NA	5.47E-10	0.00019381	0		
Tr_GCEA1	0	transport	FALSE	0	NA	6.26E-10	0.00019381	0		
Tr_GCEA2	0	transport	FALSE	0	NA	1.87E-09	0.00019381	0		
Tr_Gln	0	transport	FALSE	0	NA	9.09E-09	0.00019381	1		
Tr_Glu1	-0.619886909	transport	FALSE	0	0.630761752	5.29E-08	0.00023626	5	2.31E-15	100
Tr_Glu2	7.4545013	transport	TRUE	0	0.873843243	2.24E-08	0.00019381	6	54.19668018	100
Tr_Gly	0	transport	FALSE	0	NA	6.67E-10	0.00019381	0		
Tr_GlySer	3.395515341	transport	FALSE	1	0.926270286	1.02E-08	0.00024952	5	62.17399063	100
Tr_GPT1	0	transport	FALSE	0	0.821377643	3.56E-08	0.00025905	36	5.833147498	100
Tr_H2Oh	85.72822736	transport	FALSE	0	0.897777253	4.51E-08	0.00026617	42	40.94114228	100
Tr_H2Om	-29.32847147	transport	FALSE	0	0.891944066	1.82E-08	0.00027368	36	21.96040095	100
Tr_H2Op	0	transport	FALSE	0	NA	4.73E-10	0.00019381	0		
Tr_H2S1	-0.158067635	transport	FALSE	0	0.877933129	1.24E-08	0.00027368	21	13.79852475	100.0000002
Tr_H2S2	0	transport	FALSE	0	NA	3.66E-08	0.00023314	4		
Tr_HCys	0.170753709	transport	TRUE	1	0.948944251	5.4E-10	0.00019381	0	24.5425973	81.9919715
Tr_His1	0	transport	FALSE	0	NA	8.6E-09	0.00019381	1		
Tr_His2	0.16887082	transport	TRUE	1	0.949001023	9.3E-10	0.00019381	0	29.13142516	95.76282993
Tr_His3	0	transport	FALSE	0	NA	4.3E-09	0.00019381	1	0	0
Tr_Hp	0	transport	FALSE	0	NA	4.74E-10	0.00019381	0		
Tr_Ile1	0	transport	FALSE	0	NA	5.43E-09	0.00019381	1		
Tr_Ile2	0.456292823	transport	TRUE	1	0.950525721	4.63E-09	0.00019381	1	51.60983366	98.79987236
Tr_Ile3	0	transport	FALSE	0	NA	5.24E-10	0.00019381	0		
Tr_KG	0.619886909	transport	FALSE	0	0.671056224	4.73E-10	0.00019381	0	0	31.99284091
Tr_Leu1	0	transport	FALSE	0	NA	5.63E-10	0.00019381	0		
Tr_Leu2	0.806508612	transport	TRUE	0	0.885361162	4.81E-10	0.00019381	0	67.1464413	99.43640638
Tr_Leu3	0	transport	FALSE	0	NA	5.17E-10	0.00019381	0		
Tr_Lys1	0	transport	FALSE	0	NA	6.09E-10	0.00019381	0		
Tr_Lys2	0.526223222	transport	TRUE	0	0.896969513	9.13E-09	0.00019381	1	52.7057091	97.72056499
Tr_Mal	0	transport	FALSE	0	NA	4.51E-09	0.00019381	1		
Tr_Met1	0	transport	FALSE	0	NA	5.73E-10	0.00019381	0		
Tr_Met2	0	transport	FALSE	0	NA	6.92E-09	0.00022646	1		
Tr_Met3	0	transport	FALSE	0	NA	7.44E-09	0.00019381	1		
Tr_Mex1	0.013255864	transport	TRUE	1	0.948048362	1.2E-09	0.00019381	0	0.320691508	100
Tr_NDT1	-0.010376237	transport	FALSE	0	0.884750195	1.24E-08	0.00019381	0	0.144877412	33.39560453
Tr_NDT2	0.010376237	transport	FALSE	0	0.88256513	6.58E-10	0.00019381	0	0.196520542	35.97172332
Tr_NH41	1.68991145	transport	FALSE	0	0.79032636	4.77E-08	0.00025628	69	37.24651691	100.0000002
Tr_NH42	0.345565433	transport	FALSE	0	0.785437174	6.65E-08	0.00019381	0	1.291166637	69.00061608

**Tr_NO2**	9.36310494	transport	FALSE	0	0.890603178	7.88E-09	0.00023245	3	87.67264709	100
**Tr_NTT**	14.54295505	transport	FALSE	1	0.90172822	9.64E-09	0.00023356	2	45.67006036	100
**Tr_O2h**	-124.9844356	transport	TRUE	1	0.947584652	6.11E-09	0.00025621	1	97.78079634	100.0000001
**Tr_O2m**	4.49148085	transport	TRUE	0	0.895208981	9.6E-09	0.00019381	0	34.12439575	100
**Tr_O2p**	0	transport	FALSE	0	NA	4.31E-09	0.00019381	1		
**Tr_OAA**	0.871579473	transport	FALSE	0	0.579262892	5.14E-10	0.00019381	0	4.52E-16	6.494597615
**Tr_ORO**	0.011205395	transport	TRUE	1	0.949058635	4.56E-09	0.00019381	0	96.80152519	100
**Tr_P5C1**	0	transport	FALSE	0	NA	5.01E-08	0.00025675	35		
**Tr_P5C2**	23.82819817	transport	FALSE	0	0.893356924	9.77E-09	0.00025675	3	46.82135603	100
**Tr_pGlc**	0	transport	FALSE	0	NA	9.17E-09	0.00019381	0		
**Tr_Phe1**	0	transport	FALSE	0	NA	6.01E-10	0.00019381	0		
**Tr_Phe2**	0.338621087	transport	TRUE	1	0.948908058	5.62E-10	0.00019381	0	50.94640793	88.57391712
**Tr_Phe3**	0	transport	FALSE	0	NA	5.75E-09	0.00019381	1		
**Tr_PLT5**	0	transport	FALSE	0	NA	5.38E-09	0.00019381	0		
**Tr_PPT**	0	transport	FALSE	0	0.846380331	5.63E-10	0.00019381	0	0	24.24512376
**Tr_Pro1**	0	transport	FALSE	0	NA	5.28E-09	0.00019381	0		
**Tr_Pro2**	0	transport	FALSE	0	NA	9.29E-09	0.00019381	1		
**Tr_ProA**	0	transport	FALSE	0	0.795853136	5.26E-09	0.00019507	0	0	100
**Tr_ProU**	23.27940405	transport	FALSE	0	0.892579198	1.54E-08	0.00020691	41	53.03557139	100
**Tr_PRPP**	0.011205395	transport	TRUE	1	0.93257451	1.48E-09	0.00019381	0	0.47942817	100
**Tr_Pyr1**	-3.19479556	transport	FALSE	0	0.899998149	2.14E-08	0.00021951	21	36.36944411	100
**Tr_Pyr2**	5.302431938	transport	FALSE	0	0.897212935	1.05E-08	0.00019484	5	2.126819693	100
**Tr_Pyr3**	-1.491466382	transport	FALSE	0	0.737637669	4.41E-08	0.00019381	1	0	98.42223213
**Tr_Ser1**	0	transport	FALSE	0	NA	5.28E-10	0.00019381	0		
**Tr_Ser2**	0	transport	FALSE	0	NA	1.33E-08	0.00019381	1		
**Tr_Ser3**	1.69775767	transport	FALSE	1	0.91939514	9.57E-09	0.00023626	0	56.21253976	100
**Tr_SFC**	0	transport	FALSE	0	NA	1.9E-08	0.00019381	1		
**Tr_SO4**	0.328821344	transport	FALSE	0	0.779257692	1.57E-09	0.00019381	0	1.64E-12	100.0000002
**Tr_Thr1**	0	transport	FALSE	0	NA	4.01E-09	0.00019381	1		
**Tr_Thr2**	5.30475864	transport	TRUE	1	0.942500445	8.9E-09	0.0002541	1	73.36273098	100
**Tr_Thr3**	0	transport	FALSE	0	NA	5.5E-10	0.00019381	0		
**Tr_TPT1**	-9.332993326	transport	FALSE	0	0.806184338	2.21E-08	0.00020691	29	11.40574462	100
**Tr_TPT2**	27.05973029	transport	FALSE	0	0.87300955	2.51E-08	0.00025113	33	16.90901704	100
**Tr_TPT3**	0	transport	FALSE	0	0.859698552	2.83E-08	0.00019381	1	6.674080917	100
**Tr_Trp1**	0	transport	FALSE	0	NA	8.45E-10	0.00019381	0		
**Tr_Trp2**	0.085699644	transport	TRUE	1	0.948756464	8.55E-10	0.00019381	0	28.98650686	78.68935944
**Tr_Trp3**	0	transport	FALSE	0	NA	3.87E-09	0.00019381	1		
**Tr_Tyr1**	0	transport	FALSE	0	NA	4.39E-10	0.00019381	0		
**Tr_Tyr2**	0.238893923	transport	TRUE	1	0.949004133	5.39E-10	0.00019381	0	51.24568579	94.93237769
**Tr_Tyr3**	0	transport	FALSE	0	NA	8.87E-10	0.00019381	0		
**Tr_Val1**	0	transport	FALSE	0	NA	7.04E-09	0.00019381	1		
**Tr_Val2**	0.743000672	transport	TRUE	1	0.930273071	5.65E-09	0.00019381	1	58.06706719	93.58224503
**Tr_Val3**	0	transport	FALSE	0	NA	5.95E-10	0.00019381	0		
**TrpS_h**	0.085699644	amino acid synthesis	TRUE	0	0.890399313	1.38E-09	0.00019381	0	29.34876083	96.95409081
**TRXR_c**	0.010915192	TRX recycling	TRUE	1	0.947049066	6.63E-09	0.00022238	0	97.0926268	100
**UDPK_c**	3.957098911	nucleotide metabolism	TRUE	1	0.949571772	5.12E-09	0.00019381	1	15.41984695	78.06013746
**UDPR_c**	0.003519262	nucleotide metabolism	TRUE	1	0.948673847	1.21E-08	0.00026534	0	96.58700265	100
**UGPase_c**	3.949412778	sucrose synthesis	TRUE	1	0.949639141	4.79E-10	0.00019381	0	15.24570862	77.53066032
**Urease_m**	0	amino acid synthesis	FALSE	0	NA	6.26E-08	0.00019381	1		
